# Supplementary material for: Impact and cost-effectiveness of the 6-month BPaLM regimen for rifampicin-resistant tuberculosis in Moldova: A mathematical modeling analysis
Source: PLoS Med. 2024 May 3;21(5):e1004401. doi: 10.1371/journal.pmed.1004401 (PMC11101189; doi:10.1371/journal.pmed.1004401)
Supplement: S1 Checklist — (PDF) [file pmed.1004401.s001.pdf]

S1 Checklist. CHEERS 2022 Checklist

Topic	No.	Item	Location where item is reported
**Title**			
	1	Identify the study as an economic evaluation and specify the interventions being compared.	Title, Page 1
**Abstract**			
	2	Provide a structured summary that highlights context, key methods, results, and alternative analyses.	Abstract, Page 1
**Introduction**			
**Background and objectives**	3	Give the context for the study, the study question, and its practical relevance for decision making in policy or practice.	Introduction, Lines 2-64
**Methods**			
**Health economic analysis plan**	4	Indicate whether a health economic analysis plan was developed and where available.	Not Applicable
**Study population**	5	Describe characteristics of the study population (such as age range, demographics, socioeconomic, or clinical characteristics).	Methods, page 11, Lines 2-11; Fig S2
**Setting and location**	6	Provide relevant contextual information that may influence findings.	Introduction, page 7 Lines 53-64; Methods, page 11 line 2-11
**Comparators**	7	Describe the interventions or strategies being compared and why chosen.	Methods, page 8; Table 1
**Perspective**	8	State the perspective(s) adopted by the study and why chosen.	Methods, page 18 Line 42-page 19 line 52
**Time horizon**	9	State the time horizon for the study and why appropriate.	Methods, page 11 Line 14
**Discount rate**	10	Report the discount rate(s) and reason chosen.	Methods, page 19 Lines 53-54

Topic	No.	Item	Location where item is reported
**Selection of outcomes**	11	Describe what outcomes were used as the measure(s) of benefit(s) and harm(s).	Methods, page 17 lines 16-21, page 18 lines 34-41.
**Measurement of outcomes**	12	Describe how outcomes used to capture benefit(s) and harm(s) were measured.	Methods, page 17 line 22 – page 18 line 33. S1 Appendix, S2 Appendix; Table S1
**Valuation of outcomes**	13	Describe the population and methods used to measure and value outcomes.	Methods; S1 and S2 Appendix; Table S1
**Measurement and valuation of resources and costs**	14	Describe how costs were valued.	Methods page 18 line 42 – page 19 line 52; Table S1
**Currency, price date, and conversion**	15	Report the dates of the estimated resource quantities and unit costs, plus the currency and year of conversion.	Table 2; Table S1
**Rationale and description of model**	16	If modelling is used, describe in detail and why used. Report if the model is publicly available and where it can be accessed.	Methods, page 11 Lines 14-33; S1 Appendix
**Analytics and assumptions**	17	Describe any methods for analysing or statistically transforming data, any extrapolation methods, and approaches for validating any model used.	Methods page 20 Lines 80-94; S1 and S2 Appendix; Table S1
**Characterising heterogeneity**	18	Describe any methods used for estimating how the results of the study vary for subgroups.	Methods page 20 Lines 80-94
**Characterising distributional effects**	19	Describe how impacts are distributed across different individuals or adjustments made to reflect priority populations.	Not reported
**Characterising uncertainty**	20	Describe methods to characterise any sources of uncertainty in the analysis.	Methods page 20 Lines 80-88; S1 Appendix, Table S1
**Approach to engagement with patients and others affected by the study**	21	Describe any approaches to engage patients or service recipients, the general public, communities, or stakeholders (such as clinicians or payers) in the design of the study.	Not reported
**Results**			
**Study parameters**	22	Report all analytic inputs (such as values, ranges, references) including uncertainty or distributional assumptions.	Table 2; Table S1; Table S2; Fig S3

Topic	No.	Item	Location where item is reported
**Summary of main results**	23	Report the mean values for the main categories of costs and outcomes of interest and summarise them in the most appropriate overall measure.	Results p.22-28
**Effect of uncertainty**	24	Describe how uncertainty about analytic judgments, inputs, or projections affect findings. Report the effect of choice of discount rate and time horizon, if applicable.	Results page 28 line 97 – page 29 line 105; Fig 1B, Fig 5
**Effect of engagement with patients and others affected by the study**	25	Report on any difference patient/service recipient, general public, community, or stakeholder involvement made to the approach or findings of the study	Not reported
**Discussion**			
**Study findings, limitations, generalisability, and current knowledge**	26	Report key findings, limitations, ethical or equity considerations not captured, and how these could affect patients, policy, or practice.	Discussion
**Other relevant information**			
**Source of funding**	27	Describe how the study was funded and any role of the funder in the identification, design, conduct, and reporting of the analysis	Metadata
**Conflicts of interest**	28	Report authors conflicts of interest according to journal or International Committee of Medical Journal Editors requirements.	Metadata
