# Supplementary material for: Impact and cost-effectiveness of the 6-month BPaLM regimen for rifampicin-resistant tuberculosis in Moldova: A mathematical modeling analysis
Source: PLoS Med. 2024 May 3;21(5):e1004401. doi: 10.1371/journal.pmed.1004401 (PMC11101189; doi:10.1371/journal.pmed.1004401)
Supplement: S2 Table — LTFU, lost to follow up. LTFU data from Walker and colleagues [78]. *The values in the rightmost column are used as the model inputs. Compared to the fourth column, we rounded down the values from month 21 onwards such that the probability of LTFU is zero thenceforth. (PDF) [file pmed.1004401.s005.pdf]

S2 Table. Probability of loss to follow up by month of treatment.

Time, t, in months	Proportion LTFU at *t*	LTFU-free survival at *t*	p(LTFU by *t+1* | alive at *t*)	Adjusted* p(LTFU by *t+1* | alive at *t*)
0	0	1	0.025	0.025
1	0.0254	0.97461	0.009	0.009
2	0.0340	0.96601	0.012	0.012
3	0.0452	0.95481	0.011	0.011
4	0.0553	0.94472	0.015	0.015
5	0.0693	0.93072	0.015	0.015
6	0.0836	0.91641	0.013	0.013
7	0.0951	0.90487	0.015	0.015
8	0.1088	0.8912	0.011	0.011
9	0.1189	0.88114	0.008	0.008
10	0.1259	0.87414	0.005	0.005
11	0.1302	0.86977	0.011	0.011
12	0.1400	0.85997	0.005	0.005
13	0.1443	0.85567	0.005	0.005
14	0.1485	0.85146	0.008	0.008
15	0.1556	0.84436	0.003	0.003
16	0.1586	0.84145	0.005	0.005
17	0.1627	0.83731	0.002	0.002
18	0.1642	0.83577	0.005	0.005
19	0.1685	0.83154	0.003	0.003
20	0.1712	0.82885	0.000	0.000
21	0.1712	0.82885	0.000	0
22	0.1712	0.82882	0.001	0
23	0.1724	0.82765	0.000	0
24	0.1720	0.828	--	0

LTFU – Lost to Follow Up.

LTFU data from Walker et al. 2019 [1]. *The values in the rightmost column are used as the model inputs. Compared to the fourth column, we rounded down the values from month 21 onwards such that the probability of LTFU is zero thenceforth.

REFERENCE

This reference is provided here for convenience. It is also provided in the main manuscript file in the legend for S2 Table.

1. Walker IF, Shi O, Hicks JP, Elsey H, Wei X, Menzies D, et al. Analysis of loss to follow-up in 4099 multidrug-resistant pulmonary tuberculosis patients. Eur Respir J. 2019;54. doi:10.1183/13993003.00353-2018
