# Supplementary material for: Impact and cost-effectiveness of the 6-month BPaLM regimen for rifampicin-resistant tuberculosis in Moldova: A mathematical modeling analysis
Source: PLoS Med. 2024 May 3;21(5):e1004401. doi: 10.1371/journal.pmed.1004401 (PMC11101189; doi:10.1371/journal.pmed.1004401)
Supplement: S3 Table — BPaLM, bedaquiline, pretomanid, linezolid, moxifloxacin; SOC, standard of care. The entire cohort had RR-TB, and so the duration with rifampicin resistance is equivalent to the duration with active RR-TB, and the cumulative incidence of rifampicin resistance is not applicable. Some drugs were used very sparingly, if ever, under one or both strategies (e.g., amikacin, ethambutol, ethionamide, isoniazid, and pyrazinamide); as such the cumulative incidence may be very low for these drugs under one or both strategies. (PDF) [file pmed.1004401.s006.pdf]

S3 Table. Duration and cumulative incidence of resistance to key drugs.

Drugs	Duration with resistance, 6 months BPaLM, entire cohort (months)	Duration with resistance, SOC, entire cohort (months)	Difference in duration, BPaLM vs. SOC, entire cohort	p-value	Duration with resistance, 6 months BPaLM, active untreated TB (months)	Duration with resistance, SOC, active untreated TB (months)	Difference in duration, BPaLM vs. SOC, active untreated TB	p-value	Lifetime cumulative incidence of resistance, 6 months BPaLM (%)	Lifetime cumulative incidence of resistance, SOC (%)	Difference in cumulative incidence, BPaLM vs. SOC (p.p.)	p-value
Amikacin	1.79<br>(1.40, 2.25)	2.18<br>(1.66, 2.71)	-0.40<br>(-0.79, -0.06)	0.022	0.43<br>(0.31, 0.56)	0.42<br>(0.29, 0.55)	0.01<br>(-0.10, 0.12)	0.868	0.00<br>(0.00, 0.00)	0.00<br>(0.00, 0.02)	0.00<br>(-0.02, 0.00)	<0.001
Bedaquiline	0.93<br>(0.56, 1.47)	1.86<br>(1.29, 2.51)	-0.92<br>(-1.48, -0.49)	<0.001	0.21<br>(0.12, 0.34)	0.39<br>(0.26, 0.54)	-0.18<br>(-0.30, -0.06)	<0.001	3.13<br>(2.22, 4.33)	5.22<br>(4.07, 6.59)	-2.09<br>(-3.03, -1.24)	<0.001
Clofazimine	1.09<br>(0.66, 1.67)	2.40<br>(1.75, 3.20)	-1.31<br>(-1.94, -0.80)	<0.001	0.24<br>(0.15, 0.39)	0.50<br>(0.35, 0.68)	-0.25<br>(-0.40, -0.12)	<0.001	3.81<br>(2.71, 5.17)	7.67<br>(6.10, 9.55)	-3.86<br>(-5.05, -2.80)	<0.001
Cycloserine	0.61<br>(0.42, 0.88)	1.56<br>(1.15, 2.05)	-0.95<br>(-1.38, -0.62)	<0.001	0.14<br>(0.09, 0.21)	0.31<br>(0.22, 0.44)	-0.17<br>(-0.28, -0.09)	<0.001	0.75<br>(0.45, 1.15)	3.07<br>(2.29, 3.97)	-2.32<br>(-3.10, -1.66)	<0.001
Delamanid	1.06<br>(0.65, 1.60)	0.52<br>(0.37, 0.70)	0.54<br>(0.18, 1.04)	0.002	0.24<br>(0.14, 0.37)	0.11<br>(0.07, 0.16)	0.13<br>(0.04, 0.24)	<0.001	3.16<br>(2.20, 4.32)	1.50<br>(0.98, 2.13)	1.66<br>(0.92, 2.52)	<0.001
Ethambutol	13.48<br>(10.92, 16.50)	15.01<br>(13.75, 16.16)	-1.52<br>(-4.26, 1.45)	0.302	3.26<br>(2.58, 4.13)	2.99<br>(2.60, 3.41)	0.27<br>(-0.40, 1.02)	0.474	0.00<br>(0.00, 0.00)	0.19<br>(0.10, 0.30)	-0.19<br>(-0.30, -0.10)	<0.001
Ethionamide	9.92<br>(8.05, 12.08)	11.39<br>(10.42, 12.29)	-1.47<br>(-3.47, 0.70)	0.202	2.40<br>(1.88, 3.05)	2.28<br>(1.96, 2.59)	0.12<br>(-0.38, 0.67)	0.714	0.00<br>(0.00, 0.00)	0.37<br>(0.20, 0.56)	-0.37<br>(-0.56, -0.20)	<0.001
Isoniazid	14.76<br>(11.92, 18.09)	15.91<br>(14.57, 17.11)	-1.16<br>(-4.11, 2.18)	0.466	3.57<br>(2.81, 4.53)	3.16<br>(2.73, 3.59)	0.41<br>(-0.32, 1.23)	0.294	0.00<br>(0.00, 0.00)	0.01<br>(0.00, 0.03)	-0.01<br>(-0.03, 0.00)	<0.001
Linezolid	0.93<br>(0.64, 1.29)	1.15<br>(0.85, 1.53)	-0.22<br>(-0.54, 0.08)	0.152	0.21<br>(0.14, 0.31)	0.22<br>(0.15, 0.31)	-0.01<br>(-0.09, 0.07)	0.77	1.25<br>(0.76, 1.87)	1.15<br>(0.78, 1.57)	0.10<br>(-0.33, 0.55)	0.694
Moxifloxacin	5.41<br>(4.38, 6.73)	7.62<br>(6.66, 8.65)	-2.21<br>(-3.39, -1.02)	<0.001	1.29<br>(0.99, 1.65)	1.39<br>(1.15, 1.65)	-0.10<br>(-0.40, 0.22)	0.548	1.72<br>(1.08, 2.50)	1.47<br>(0.94, 2.09)	0.25<br>(-0.19, 0.80)	0.354
Pretomanid	1.06<br>(0.65, 1.60)	0.51<br>(0.36, 0.69)	0.55<br>(0.20, 1.05)	<0.001	0.24<br>(0.14, 0.37)	0.11<br>(0.07, 0.16)	0.13<br>(0.04, 0.24)	<0.001	3.16<br>(2.20, 4.32)	1.40<br>(0.92, 1.98)	1.76<br>(1.01, 2.66)	<0.001
Pyrazinamide	8.54<br>(6.92, 10.41)	10.83<br>(9.84, 11.84)	-2.28<br>(-4.02, -0.52)	0.016	2.06<br>(1.60, 2.59)	2.12<br>(1.82, 2.44)	-0.06<br>(-0.51, 0.40)	0.758	0.00<br>(0.00, 0.00)	0.66<br>(0.40, 0.97)	-0.66<br>(-0.97, -0.40)	<0.001
Rifampicin	14.88<br>(12.03, 18.23)	15.98<br>(14.63, 17.19)	-1.10<br>(-4.07, 2.28)	0.486	3.60<br>(2.84, 4.57)	3.17<br>(2.74, 3.61)	0.43<br>(-0.31, 1.27)	0.28	N/A	N/A	N/A	N/A

BPaLM, bedaquiline, pretomanid, linezolid, moxifloxacin; SOC, standard of care.

The entire cohort had RR-TB, and so the duration with rifampicin resistance is equivalent to the duration with active RR-TB, and the cumulative incidence of rifampicin resistance is not applicable. Some drugs were used very sparingly, if ever, under one or both strategies (e.g., amikacin, ethambutol, ethionamide, isoniazid, and pyrazinamide); as such the cumulative incidence may be very low for these drugs under one or both strategies.
