# Supplementary material for: Impact and cost-effectiveness of the 6-month BPaLM regimen for rifampicin-resistant tuberculosis in Moldova: A mathematical modeling analysis
Source: PLoS Med. 2024 May 3;21(5):e1004401. doi: 10.1371/journal.pmed.1004401 (PMC11101189; doi:10.1371/journal.pmed.1004401)
Supplement: S4 Table — BPaL, bedaquiline, pretomanid, linezolid; BPaLC, bedaquiline, pretomanid, linezolid, clofazimine; BPaLM, bedaquiline, pretomanid, linezolid, moxifloxacin; UI, uncertainty interval. Strategies are listed in the same order as Table 3. Mean values are shown with accompanying 95% UIs in parentheses. (PDF) [file pmed.1004401.s007.pdf]

S4 Table. Life Years achieved under each RR-TB treatment strategy.

Strategy	Under BPaLM, alternative regimen if Moxifloxacin stopped	DST for second-line drugs at treatment initiation	Frequency of DST during subsequent treatment course	Undiscounted Life Years
5) 6 months BPaLM	BPaLC	No	Every 4 months	14.745 (12.72, 16.55)
1) 6 months BPaLM	BPaLC	Yes	Every 4 months	14.750 (12.76, 16.54)
2) 6 months BPaLM	BPaLC	Yes	Monthly	14.753 (12.74, 16.49)
6) 6 months BPaLM	BPaL only	No	Every 4 months	14.405 (12.44, 16.10)
3) 6 months BPaLM	BPaL only	Yes	Every 4 months	14.414 (12.42, 16.17)
4) 6 months BPaLM	BPaL only	Yes	Monthly	14.408 (12.42, 16.16)
7) standard of care	--	Yes	Every 4 months	14.832 (13.00, 16.54)
8) standard of care	--	Yes	Monthly	14.836 (12.98, 16.56)

BPaL – bedaquiline, pretomanid, linezolid; BPaLC – bedaquiline, pretomanid, linezolid, clofazimine; BPaLM – bedaquiline, pretomanid, linezolid, moxifloxacin; UI – Uncertainty Interval.

Strategies are listed in the same order as Table 3. Mean values are shown with accompanying 95% UIs in parentheses.
