# Supplementary material for: Impact and cost-effectiveness of the 6-month BPaLM regimen for rifampicin-resistant tuberculosis in Moldova: A mathematical modeling analysis
Source: PLoS Med. 2024 May 3;21(5):e1004401. doi: 10.1371/journal.pmed.1004401 (PMC11101189; doi:10.1371/journal.pmed.1004401)
Supplement: S5 Table — BPaLC, bedaquiline, pretomanid, linezolid, clofazimine; BPaLM, bedaquiline, pretomanid, linezolid, moxifloxacin; LYs, life years; Mfx, moxifloxacin; p.p., percentage points; QALYs, quality-adjusted life years; UI, uncertainty interval; WHO, World Health Organization. The following health outcomes are shown: a composite “Unfavorable outcome” closely aligned to the composite trial endpoint in TB-PRACTECAL, true cure, and quality-adjusted life expectancy. Results are shown separately over 3 model-run time horizons: 6 months, 72 weeks (in line with the endpoint in TB-PRACTECAL), and lifetime (in line with the primary outcomes in our analysis). (PDF) [file pmed.1004401.s008.pdf]

S5 Table. Comparing outcomes at 6 months, 12 months, and 72 weeks from treatment initiation.

STRATEGY DETAILS				RISK OF UNFAVORABLE OUTCOMES			
**Strategy Name**	**Alternative regimen if Mfx stopped**	**2nd-line DST at treatment initiation**	**Routine frequency of subsequent 2nd-line DST**	**Based on TB-PRACTECAL trial definition**		**Based on WHO End of Treatment definition**	
				Proportion (%)	Risk Difference (p.p.)	Proportion (%)	Risk Difference (p.p.)
6 months from treatment initiation							
(7) Standard of Care	--	Yes	Every 4 months	32.9 (30.1, 36)	--	29.6 (27, 32.6)	--
(1) 6 months BPaLM	BPaLC	Yes	Every 4 months	30.9 (27.7, 34.5)	-1.9 (-5.2, 1.4) p=0.234	17.8 (16, 20)	-11.8 (-14.6, -9.4) p=<0.001
12 months from treatment initiation							
(7) Standard of Care	--	Yes	Every 4 months	39.0 (36.3, 42.2)		35.8 (33.1, 38.9)	
(1) 6 months BPaLM	BPaLC	Yes	Every 4 months	36.1 (32.6, 39.8)	-2.9 (-5.8, 0.2) p=0.068	22.8 (20.4, 25.4)	-12.9 (-15.6, -10.5) p=<0.001
17 months (i.e., 72 weeks) from treatment initiation							
(7) Standard of Care	--	Yes	Every 4 months	41.8 (39.1, 45.1)		38.0 (35.1, 41.3)	
(1) 6 months BPaLM	BPaLC	Yes	Every 4 months	38.7 (35.2, 42.5)	-3.1 (-6.0, 0.0) p=0.050	24.5 (21.9, 27.5)	-13.4 (-16.1, -11.0) p=<0.001

BPaLC – bedaquiline, pretomanid, linezolid, clofazimine; BPaLM – bedaquiline, pretomanid, linezolid, moxifloxacin; LYs – Life Years; Mfx – moxifloxacin; p.p. – percentage points; QALYs – Quality-adjusted Life Years; UI – Uncertainty Interval; WHO – World Health Organization

The outcomes of "unfavorable outcome" was closely aligned to the composite trial endpoint in TB-PRACTECAL. Results are shown separately over three model-run time horizons: 6 months, 12 months, and 72 weeks (in line with the endpoint in TB-PRACTECAL).
