# Supplementary material for: Impact and cost-effectiveness of the 6-month BPaLM regimen for rifampicin-resistant tuberculosis in Moldova: A mathematical modeling analysis
Source: PLoS Med. 2024 May 3;21(5):e1004401. doi: 10.1371/journal.pmed.1004401 (PMC11101189; doi:10.1371/journal.pmed.1004401)
Supplement: S6 Table — TB, tuberculosis. The budget impact was estimated for 6 months BPaLM (Strategy (1)) as compared to standard of care (Strategy (7)). (PDF) [file pmed.1004401.s009.pdf]

S6 Table. Five-year budget impact of implementing 6 months of BPaLM in Moldova.

**Budget Year**	**TB Program Budget Category**				
	**Drugs**	**Laboratory Tests**	**Routine Care**	**Nonroutine Care**	**Total**
Year 1	-583,795<br>(-1,275,027, -96,331)<br>p=0.002	-842<br>(-2,946, 523)<br>p=0.282	-47,267<br>(-106,039, -10,296)<br>p=0.002	380<br>(-738, 1,902)<br>p=0.588	-631,524<br>(-1,352,836, -115,576)<br>p=0.002
Year 2	-1,271,079<br>(-2,780,357, -213,592)<br>p=0.002	-2,206<br>(-8,253, 2,182)<br>p=0.36	-122,422<br>(-288,184, -24,477)<br>p=0.002	391<br>(-1,719, 3,147)<br>p=0.786	-1,395,316<br>(-3,008,507, -254,755)<br>p=0.002
Year 3	-1,475,708<br>(-3,254,882, -249,633)<br>p=0.002	-3,328<br>(-12,732, 3,751)<br>p=0.396	-159,279<br>(-395,607, -23,918)<br>p=0.006	-1<br>(-2,287, 2,689)<br>p=0.93	-1,638,316<br>(-3,532,616, -299,848)<br>p=0.002
Year 4	-1,529,185<br>(-3,367,264, -253,873)<br>p=0.002	-4,283<br>(-15,682, 4,332)<br>p=0.364	-172,308<br>(-445,490, -19,667)<br>p=0.014	-186<br>(-2,543, 2,525)<br>p=0.802	-1,705,962<br>(-3,694,619, -313,741)<br>p=0.002
Year 5	-1,555,715<br>(-3,421,880, -260,667)<br>p=0.002	-5,044<br>(-17,929, 4,367)<br>p=0.326	-183,809<br>(-485,833, -18,711)<br>p=0.024	-306<br>(-2,693, 2,395)<br>p=0.722	-1,744,873<br>(-3,794,268, -321,588)<br>p=0.002
**Total**	-6,415,481<br>(-14,173,208, -1,077,963)<br>p=0.002	-15,704<br>(-57,639, 14,895)<br>p=0.354	-685,085<br>(-1,716,138, -97,308)<br>p=0.006	278<br>(-9,970, 12,677)<br>p=0.98	-7,115,992<br>(-15,396,823, -1,301,637)<br>p=0.002

TB – tuberculosis.

The budget impact was estimated for 6 months BPaLM (Strategy (1)) as compared to standard of care (Strategy (7))
