# Supplementary material for: Impact and cost-effectiveness of the 6-month BPaLM regimen for rifampicin-resistant tuberculosis in Moldova: A mathematical modeling analysis
Source: PLoS Med. 2024 May 3;21(5):e1004401. doi: 10.1371/journal.pmed.1004401 (PMC11101189; doi:10.1371/journal.pmed.1004401)
Supplement: S1 Fig — Both standard of care strategies (Strategy 7 and Strategy 8) are modeled on the recommended workup and regimen selection in the 2020 WHO guidelines on the treatment of drug-resistant tuberculosis [2]. We assumed that DST results (by MGIT) are available in 2 weeks. *While we include the BPaL regimen as per the guidelines, no patients actually met the criteria to receive it under the standard of care (Strategies 7 and 8) in our model (i.e., in all model simulations, it is possible to adopt a WHO longer regimen). BPaL, bedaquiline, pretomanid, linezolid; DST, drug susceptibility test; FQ, fluoroquinolone; MGIT, mycobacterial growth indicator tube; WHO, World Health Organization. (PDF) [file pmed.1004401.s010.pdf]

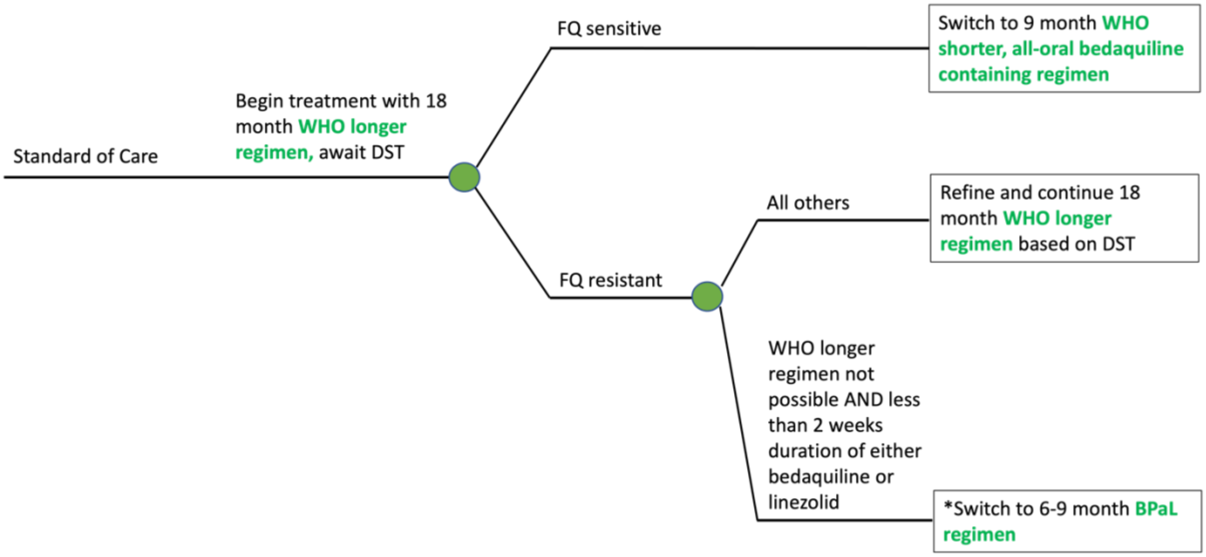

S1 Fig. Schematic of the initial workup phase for the standard of care

Both standard of care strategies (Strategy 7 and Strategy 8) are modeled on the recommended workup and regimen selection in the 2020 WHO guidelines on the treatment of drug-resistant tuberculosis [1]. We assumed that DST results (by MGIT) are available in 2 weeks. *While we include the BPaL regimen as per the guidelines, no patients actually met the criteria to receive it under the standard of care (Strategies 7 and 8) in our model (i.e., in all model simulations, it is possible to adopt a WHO longer regimen). BPaL – bedaquiline, pretomanid, linezolid; DST – drug susceptibility test; FQ – fluoroquinolone; MGIT – Mycobacterial Growth Indicator Tube; WHO – World Health Organization

REFERENCE

This references is provided here for convenience. It are also cited within the main manuscript file in the legend for S1 Fig.

1. WHO Consolidated Guidelines on Tuberculosis, Module 4: Treatment - Drug-Resistant Tuberculosis Treatment. World Health Organization; 2020. Available: https://www.who.int/publications-detail-redirect/9789240007048
