# Supplementary material for: Impact and cost-effectiveness of the 6-month BPaLM regimen for rifampicin-resistant tuberculosis in Moldova: A mathematical modeling analysis
Source: PLoS Med. 2024 May 3;21(5):e1004401. doi: 10.1371/journal.pmed.1004401 (PMC11101189; doi:10.1371/journal.pmed.1004401)
Supplement: S2 Fig — *Specimens demonstrating polyclonal infections were already excluded (n = 386), leaving a full dataset of 1,834 M. tb isolates. Exclusions made to the genomic sequencing drug susceptibility testing dataset are shown along with the number of observations. This dataset of pretreatment isolates is described elsewhere [30,35]. The presence of a mutation conferring resistance to rifampicin was assumed to convey full resistance and vice versa. The dataset with exclusion criteria applied is available at https://github.com/lyndonpjames/BPaLM_Moldova/blob/main/tbl_WGS_allRR.csv while original publicly available datasets can be found at https://www.ncbi.nlm.nih.gov/biosample?Db=biosample&DbFrom=bioproject&Cmd=Link&LinkName=bioproject_biosample&LinkReadableName=BioSample&ordinalpos=1&IdsFromResult=736718 [93] and https://www.ncbi.nlm.nih.gov/pmc/articles/PMC8903246/bin/pmed.1003933.s002.csv [30]. TB, tuberculosis. (PDF) [file pmed.1004401.s011.pdf]

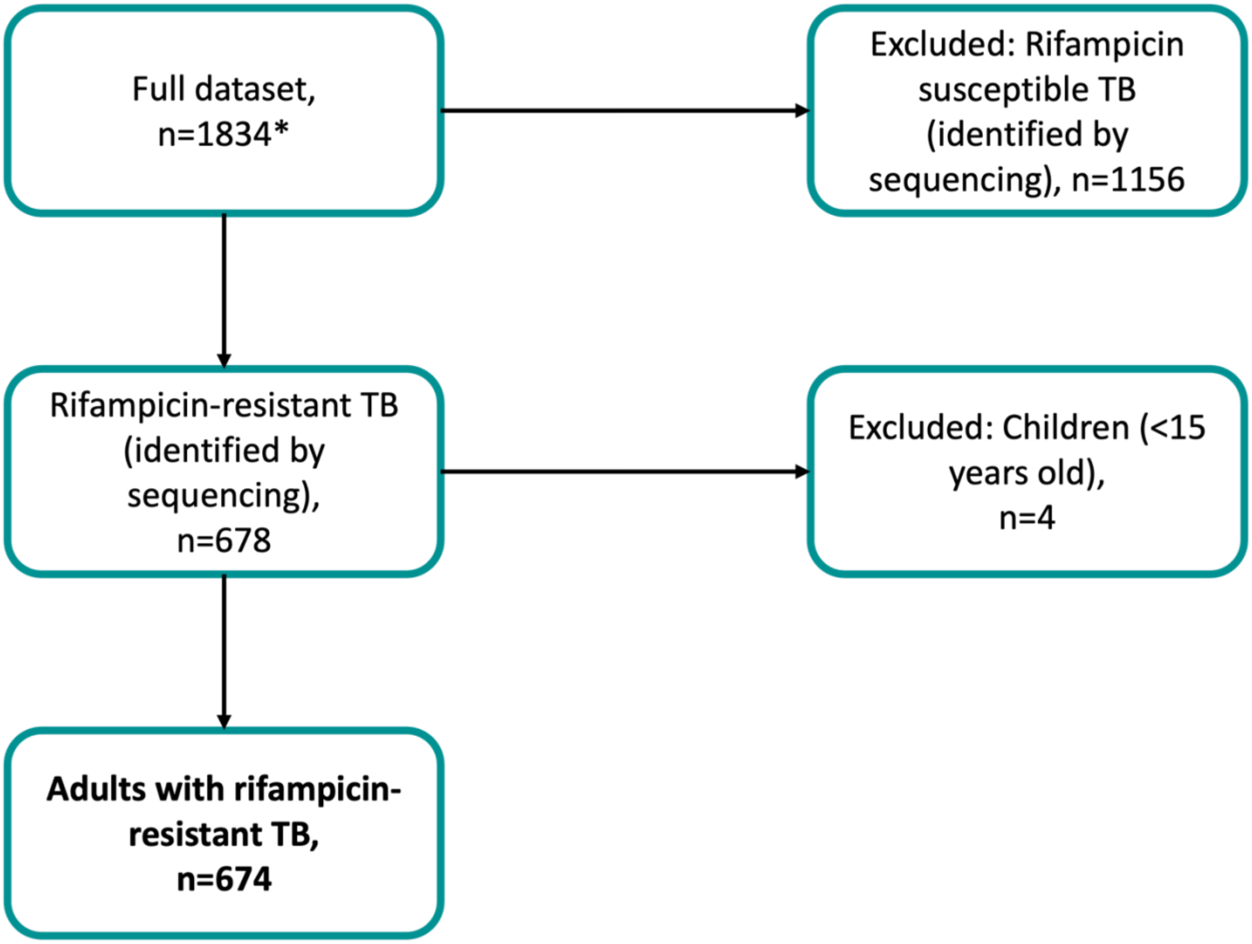

S2 Fig. ***M. tb.* genomic sequencing data exclusion criteria.**

*Specimens demonstrating polyclonal infections were already excluded (n = 386), leaving a full dataset of 1,834 *M. tb* isolates.

Exclusions made to the genomic sequencing drug susceptibility testing dataset are shown along with the number of observations. This dataset of pretreatment isolates is described elsewhere [1,2]. The presence of a mutation conferring resistance to rifampicin was assumed to convey full resistance, and vice versa. The dataset with exclusion criteria applied is available at https://github.com/lyndonpjames/BPaLM_Moldova/blob/main/tbl_WGS_allRR.csv while original publicly available datasets can be found at https://www.ncbi.nlm.nih.gov/biosample?Db=biosample&DbFrom=bioproject&Cmd=Link&LinkName=

bioproject_biosample&LinkReadableName=BioSample&ordinalpos=1&IdsFromResult=736718 [3] and

https://www.ncbi.nlm.nih.gov/pmc/articles/PMC8903246/bin/pmed.1003933.s002.csv [2]

TB – tuberculosis

REFERENCES

These references are provided here for convenience. They are also cited within the main manuscript file in the legend for S2 Fig.

1. ID 736718 - BioProject - NCBI. [cited 9 Feb 2023]. Available: https://www-ncbi-nlm-nih-gov.ezp-prod1.hul.harvard.edu/bioproject/PRJNA736718

2. Yang C, Sobkowiak B, Naidu V, Codreanu A, Ciobanu N, Gunasekera KS, et al. Phylogeography and transmission of M. tuberculosis in Moldova. 2021 Jul p. 2021.06.30.21259748. doi:10.1101/2021.06.30.21259748

3. BioSamples from BioProject PRJNA736718. National Center for Biotechnology Information; Available: https://www.ncbi.nlm.nih.gov/biosample?Db=biosample&DbFrom=bioproject&Cmd=Link&LinkName=bioproject_biosample&LinkReadableName=BioSample&ordinalpos=1&IdsFromResult=736718
