# Supplementary material for: Impact and cost-effectiveness of the 6-month BPaLM regimen for rifampicin-resistant tuberculosis in Moldova: A mathematical modeling analysis
Source: PLoS Med. 2024 May 3;21(5):e1004401. doi: 10.1371/journal.pmed.1004401 (PMC11101189; doi:10.1371/journal.pmed.1004401)
Supplement: S3 Fig — The modeled point estimate for the monthly rate that an individual’s strain of M. tuberculosis will acquire resistance to each effective drug it is exposed to is plotted, conditional on that individual beginning the month with n effective drugs in the regimen (x-axis). Estimates for 1, 3, and 4 effective drugs were obtained from the literature. The estimate for 2 drugs was calculated, assuming an additive risk (i.e., the increase in risk for 2 effective drugs compared to 3 is the same as the increase in risk for 3 effective drugs compared to 4). See also S1 Table. (PDF) [file pmed.1004401.s012.pdf]

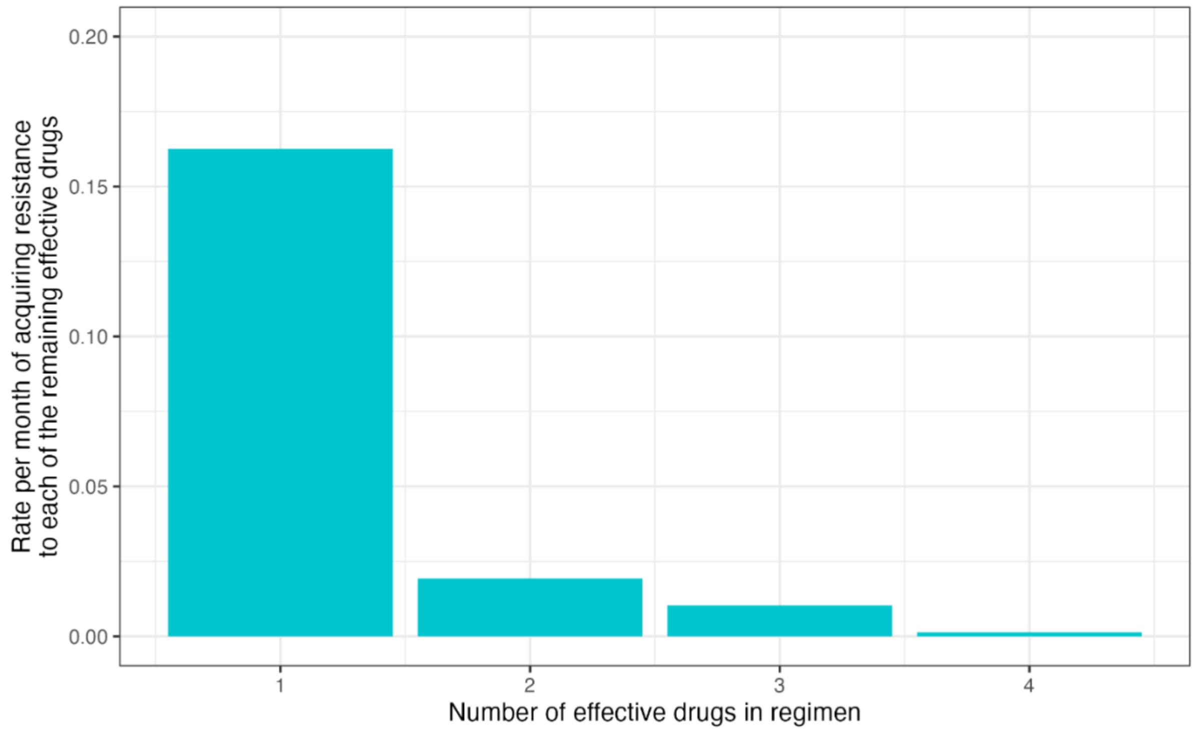

S3 Fig. The rate of acquiring drug resistance.

The modeled point estimate for the monthly rate that an individual's strain of *M. tuberculosis* will acquire resistance to each effective drug it is exposed to is plotted, conditional on that individual beginning the month with *n* effective drugs in the regimen (x-axis). Estimates for 1, 3 and 4 effective drugs were obtained from the literature. The estimate for 2 drugs was calculated, assuming an additive risk (i.e., the increase in risk for 2 effective drugs compared to 3 is the same as the increase in risk for 3 effective drugs compared to 4). See also S1 Table.
