# Supplementary material for: Impact and cost-effectiveness of the 6-month BPaLM regimen for rifampicin-resistant tuberculosis in Moldova: A mathematical modeling analysis
Source: PLoS Med. 2024 May 3;21(5):e1004401. doi: 10.1371/journal.pmed.1004401 (PMC11101189; doi:10.1371/journal.pmed.1004401)
Supplement: S4 Fig — Transitions between states can occur as shown by the arrows. Though not receiving treatment, individuals in the “Active TB, no longer receiving treatment” state are subject to a low rate of self-cure, and so may still transition to the “Cured post-treatment” state. Asterisks (*) highlight the major mechanisms through which the choice of treatment intervention affects outcomes. LTFU, lost to follow-up; TB, tuberculosis. Images within this figure were obtained as icons from Microsoft with no license or terms of use: https://support.microsoft.com/en-us/office/insert-icons-in-microsoft-365-e2459f17-3996-4795-996e-b9a13486fa79?ui=en-us&rs=en-us&ad=us. (PDF) [file pmed.1004401.s013.pdf]

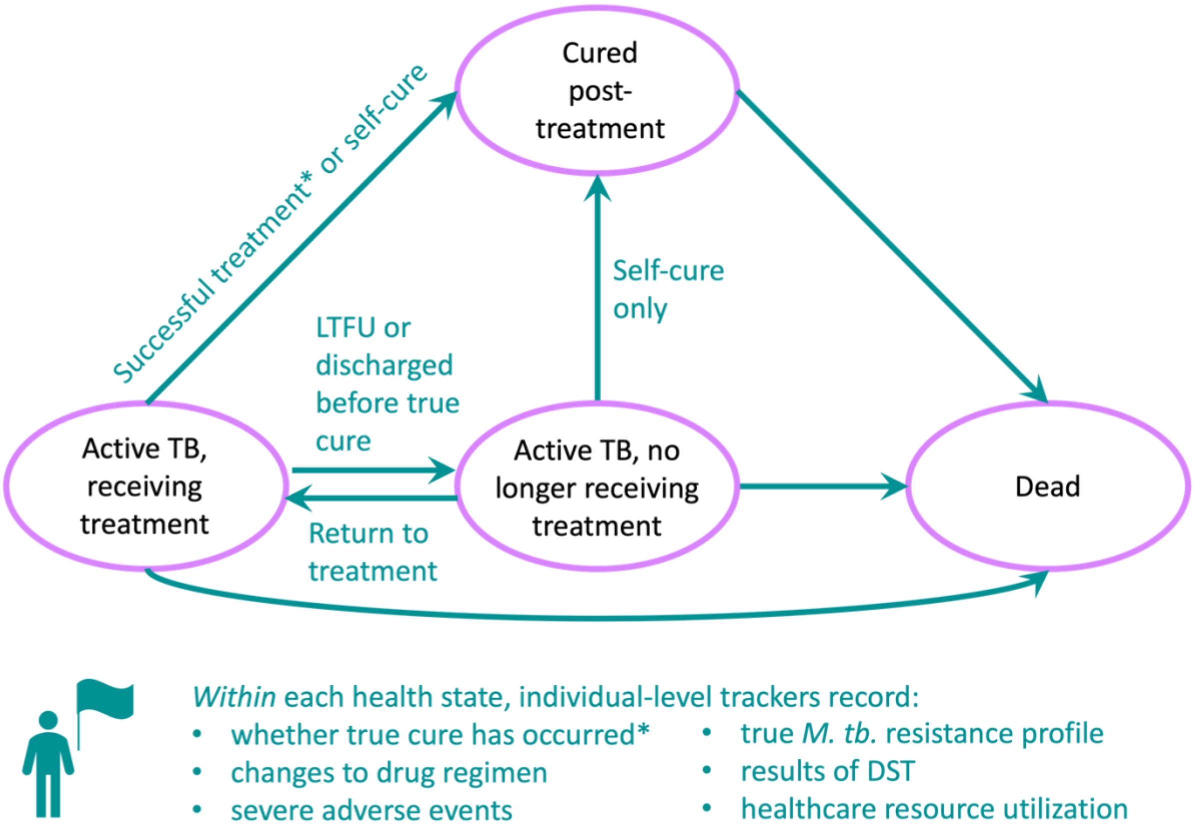

S4 Fig. Markov state-transition diagram.

Transitions between states can occur as shown by the arrows. Though not receiving treatment, individuals in the "Active TB, no longer receiving treatment" state are subject to a low rate of self-cure, and so may still transition to the "Cured post-treatment" state. Asterisks (*) highlight the major mechanisms through which the choice of treatment intervention affects outcomes. LTFU – Lost to follow-up; TB – tuberculosis.

Images within this figure were obtained as icons from Microsoft with no license or terms of use: https://support.microsoft.com/en-us/office/insert-icons-in-microsoft-365-e2459f17-3996-4795-996e-b9a13486fa79?ui=en-us&rs=en-us&ad=us
