# Supplementary material for: Impact and cost-effectiveness of the 6-month BPaLM regimen for rifampicin-resistant tuberculosis in Moldova: A mathematical modeling analysis
Source: PLoS Med. 2024 May 3;21(5):e1004401. doi: 10.1371/journal.pmed.1004401 (PMC11101189; doi:10.1371/journal.pmed.1004401)
Supplement: S5 Fig — The proportion of the cohort with primary resistance to each drug is plotted, as described by M. tuberculosis whole genomic sequencing data from Moldova [30,35]. All those observations with rifampicin susceptibility were excluded, as per S2 Fig. *There was no resistance data for pretomanid; resistance was assumed to be at the same level as for delamanid. (PDF) [file pmed.1004401.s014.pdf]

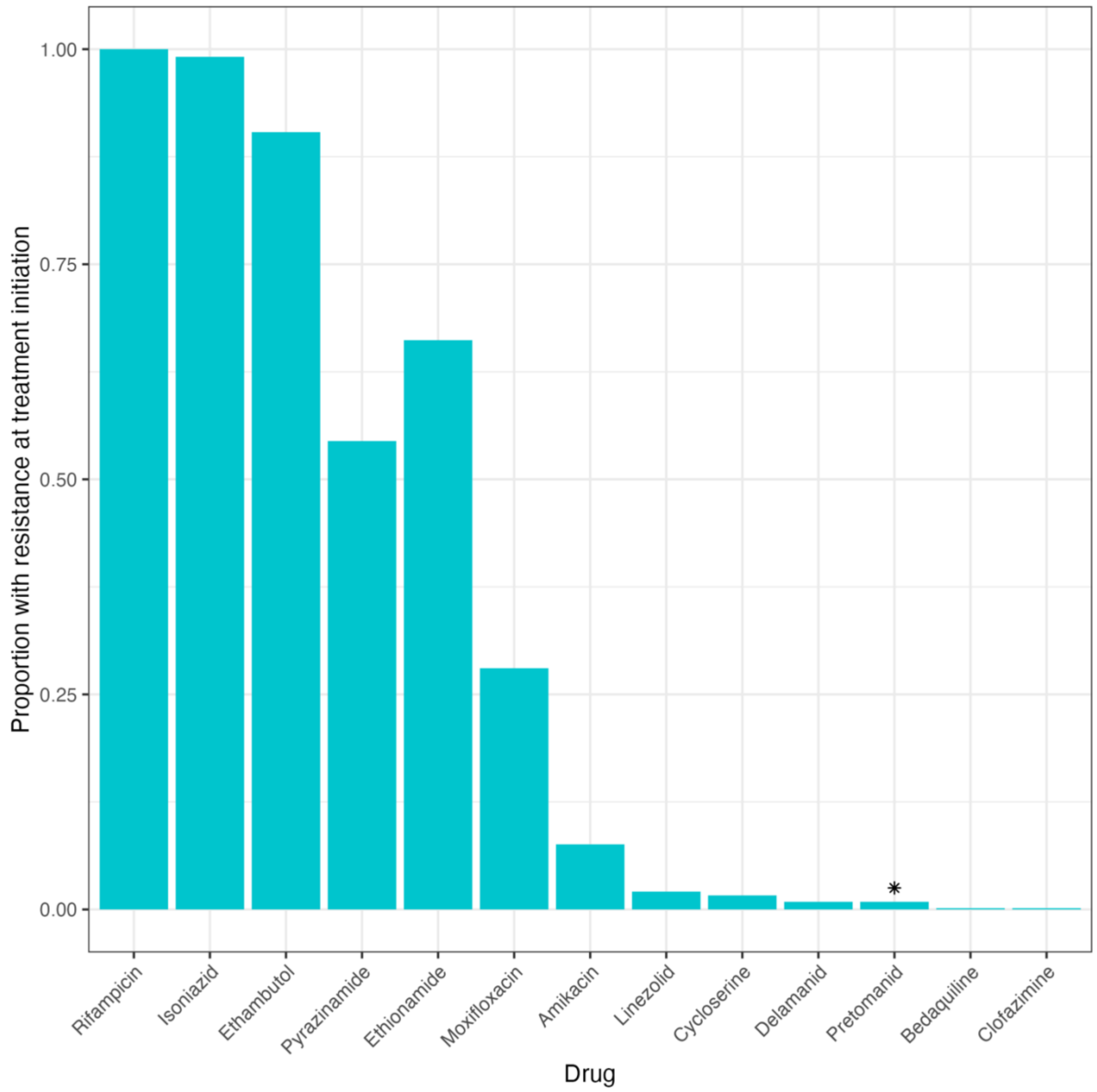

S5 Fig. Cohort prevalence of *M. tb.* resistance to key drugs at treatment initiation.

The proportion of the cohort with primary resistance to each drug is plotted, as described by *M. tuberculosis* whole genomic sequencing data from Moldova [1,2]. All those observations with rifampicin susceptibility were excluded, as per S3 Fig. *There was no resistance data for pretomanid; resistance was assumed to be at the same level as for delamanid.

REFERENCES

These references are provided here for convenience. They are also cited within the main manuscript file in the legend for S5 Fig.

1. ID 736718 - BioProject - NCBI. [date accessed: 9 Feb 2023]. Available: https://www-ncbi-nlm-nih-gov.ezp-prod1.hul.harvard.edu/bioproject/PRJNA736718

2. Yang C, Sobkowiak B, Naidu V, Codreanu A, Ciobanu N, Gunasekera KS, et al. Phylogeography and transmission of M. tuberculosis in Moldova. 2021 Jul p. 2021.06.30.21259748. doi:10.1101/2021.06.30.21259748
