# Supplementary material for: Impact and cost-effectiveness of the 6-month BPaLM regimen for rifampicin-resistant tuberculosis in Moldova: A mathematical modeling analysis
Source: PLoS Med. 2024 May 3;21(5):e1004401. doi: 10.1371/journal.pmed.1004401 (PMC11101189; doi:10.1371/journal.pmed.1004401)
Supplement: S7 Fig — The incremental cost-effectiveness plane compares the incremental discounted total QALYs and incremental discounted total costs for Strategy (1) as compared to a reference of Strategy (7). Each light pink point represents 1 iteration of the second-order Monte Carlo simulation, itself an average of 10,000 individual patient simulations. The purple diamond is the mean of the 1,000 second-order Monte Carlo simulations, corresponding to the point estimates in Table 3. The blue dot represents the standard of care (Strategy (7)), which is the reference point. BPaL, bedaquiline, pretomanid, linezolid; BPaLC, bedaquiline, pretomanid, linezolid, clofazimine; BPaLM, bedaquiline, pretomanid, linezolid, moxifloxacin; FQ-R, fluoroquinolone-resistant; FQ-S, fluoroquinolone-susceptible; Mfx, moxifloxacin; QALY, quality-adjusted life year; USD, United States dollars; WTP, willingness-to-pay. (PDF) [file pmed.1004401.s016.pdf]

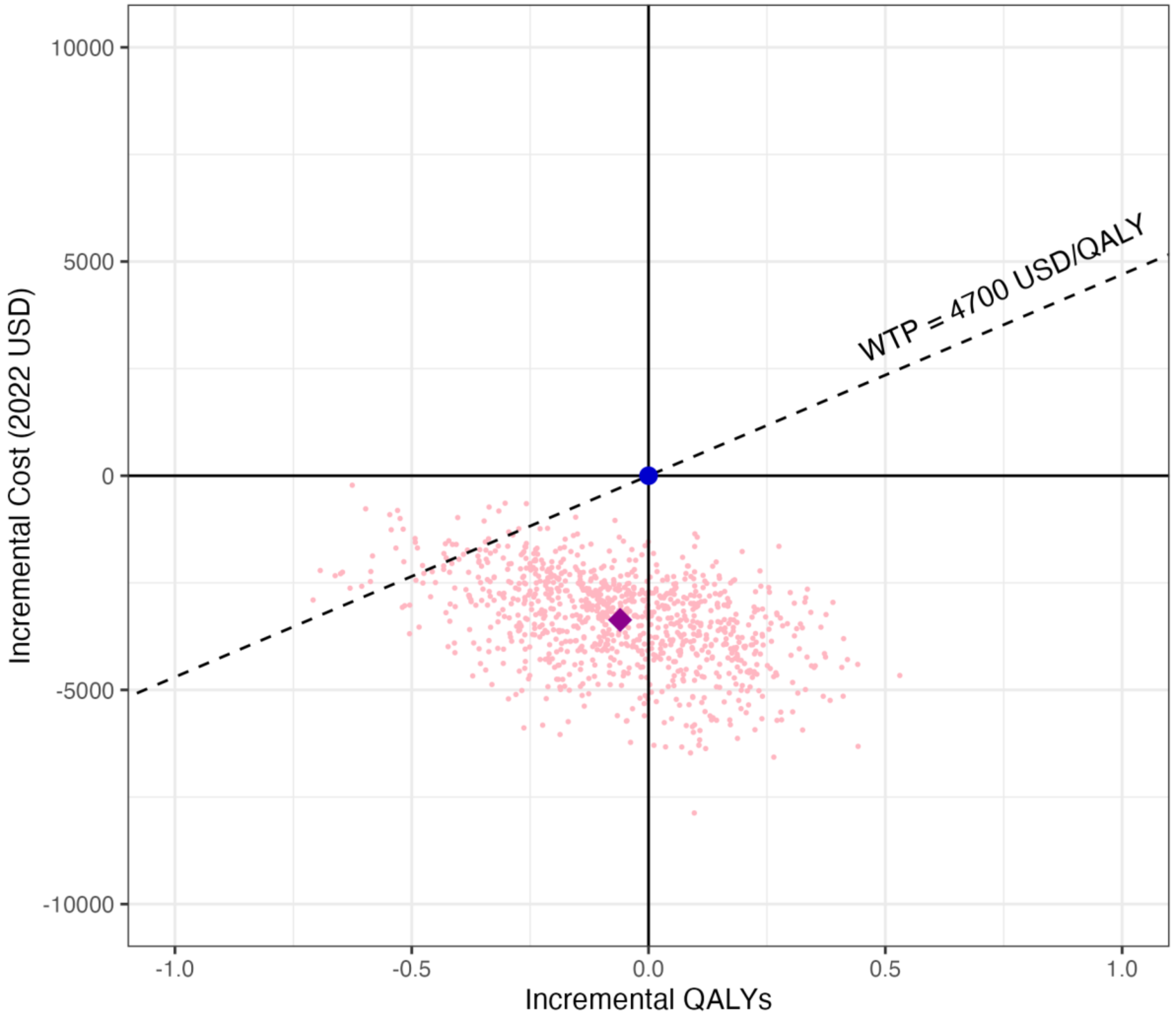

S7 Fig. Incremental cost-effectiveness plane for the leading 6-month BPaLM strategy vs. the leading SOC strategy.

The incremental cost-effectiveness plane compares the incremental discounted total QALYs and incremental discounted total costs for Strategy (1) as compared to a reference of Strategy (7). Each light pink point represents one iteration of the second-order Monte Carlo simulation, itself an average of 10,000 individual patient simulations. The purple diamond is the mean of the 1,000 second-order Monte Carlo simulations, corresponding to the point estimates in Table 3. The blue dot represents the standard of care (Strategy (7)), which is the reference point. BPaL – bedaquiline, pretomanid, linezolid; BPaLC – bedaquiline, pretomanid, linezolid, clofazimine; BPaLM – bedaquiline, pretomanid, linezolid,

moxifloxacin; FQ-R – fluoroquinolone-resistant; FQ-S – fluoroquinolone-susceptible; Mfx – moxifloxacin; QALY – quality-adjusted life year; USD – United States dollars; WTP – willingness-to-pay
