# Supplementary material for: Impact and cost-effectiveness of the 6-month BPaLM regimen for rifampicin-resistant tuberculosis in Moldova: A mathematical modeling analysis
Source: PLoS Med. 2024 May 3;21(5):e1004401. doi: 10.1371/journal.pmed.1004401 (PMC11101189; doi:10.1371/journal.pmed.1004401)
Supplement: Attachment — Submitted filename: PLOSMed_ResponsetoReviewers_20240123.docx [file pmed.1004401.s019.docx]

*Comments of the academic editor and the editorial team for your Manuscript (PMEDICINE-D-23-02158R1)* 

*Overall, the disparity between this study and the TB-PRACTECAL trial will be important for them to address, particularly in a study that relies on modelling. Also, the justification of the 2 assumptions that were questioned by the stats reviewer (ie, no mixed infections and and that a mutation associated with resistance conferred full resistance to the drug) seems quite important, as well as the potential impact of secondary transmission (due to mistreated/untreated cases of RR-TB).*

*In light of these reviews, I am afraid that we will not be able to accept the manuscript for publication in the journal in its current form, but we would like to consider a revised version that addresses the reviewers' and editors' comments. Obviously we cannot make any decision about publication until we have seen the revised manuscript and your response, and we plan to seek re-review by one or more of the reviewers. 

In revising the manuscript for further consideration, your revisions should address the specific points made by each reviewer and the editors. Please also check the guidelines for revised papers at* [*http://journals.plos.org/plosmedicine/s/revising-your-manuscript*](http://journals.plos.org/plosmedicine/s/revising-your-manuscript) *for any that apply to your paper. In your rebuttal letter you should indicate your response to the reviewers' and editors' comments, the changes you have made in the manuscript, and include either an excerpt of the revised text or the location (eg: page and line number) where each change can be found. Please submit a clean version of the paper as the main article file; a version with changes marked should be uploaded as a marked up manuscript.

In addition, we request that you upload any figures associated with your paper as individual TIF or EPS files with 300dpi resolution at resubmission; please read our figure guidelines for more information on our requirements:* [*http://journals.plos.org/plosmedicine/s/figures*](http://journals.plos.org/plosmedicine/s/figures)*. While revising your submission, please upload your figure files to the PACE digital diagnostic tool,* [*https://pacev2.apexcovantage.com/*](https://pacev2.apexcovantage.com/)*. PACE helps ensure that figures meet PLOS requirements. To use PACE, you must first register as a user. Then, login and navigate to the UPLOAD tab, where you will find detailed instructions on how to use the tool. If you encounter any issues or have any questions when using PACE, please email us at* [*PLOSMedicine@plos.org*](mailto:PLOSMedicine@plos.org)*.

We expect to receive your revised manuscript by Nov 13 2023 11:59PM. Please email us (*[*plosmedicine@plos.org*](mailto:plosmedicine@plos.org)*) if you have any questions or concerns.

***Please note while forming your response, if your article is accepted, you may have the opportunity to make the peer review history publicly available. The record will include editor decision letters (with reviews) and your responses to reviewer comments. If eligible, we will contact you to opt in or out.***

We ask every co-author listed on the manuscript to fill in a contributing author statement, making sure to declare all competing interests. If any of the co-authors have not filled in the statement, we will remind them to do so when the paper is revised. If all statements are not completed in a timely fashion this could hold up the re-review process. If new competing interests are declared later in the revision process, this may also hold up the submission. Should there be a problem getting one of your co-authors to fill in a statement we will be in contact.  YOU MUST NOT ADD OR REMOVE AUTHORS UNLESS YOU HAVE ALERTED THE EDITOR HANDLING THE MANUSCRIPT TO THE CHANGE AND THEY SPECIFICALLY HAVE AGREED TO IT. You can see our competing interests policy here:* [*http://journals.plos.org/plosmedicine/s/competing-interests*](http://journals.plos.org/plosmedicine/s/competing-interests)*.

Please use the following link to submit the revised manuscript:*

[*https://www.editorialmanager.com/pmedicine/*](https://www.editorialmanager.com/pmedicine/) *Your article can be found in the "Submissions Needing Revision" folder.

To enhance the reproducibility of your results, we recommend that you deposit your laboratory protocols in* [*protocols.io*](http://protocols.io/)*, where a protocol can be assigned its own identifier (DOI) such that it can be cited independently in the future. Additionally, PLOS ONE offers an option to publish peer-reviewed clinical study protocols. Read more information on sharing protocols at* [*https://plos.org/protocols?utm_medium=editorial-email&utm_source=authorletters&utm_campaign=protocols*](https://plos.org/protocols?utm_medium=editorial-email&utm_source=authorletters&utm_campaign=protocols) *Please ensure that the paper adheres to the PLOS Data Availability Policy (see* [*http://journals.plos.org/plosmedicine/s/data-availability*](http://journals.plos.org/plosmedicine/s/data-availability)*), which requires that all data underlying the study's findings be provided in a repository or as Supporting Information. For data residing with a third party, authors are required to provide instructions with contact information for obtaining the data. PLOS journals do not allow statements supported by "data not shown" or "unpublished results." For such statements, authors must provide supporting data or cite public sources that include it.

We look forward to receiving your revised manuscript.

Sincerely,

Katrien Janin, PhD
PLOS Medicine*
[*plosmedicine.org*](http://plosmedicine.org/) *-----------------------------------------------------------
Requests from the editors:

GENERAL: Please provide 95% UIs and p values for all results were appropriate (including the abstract), check and amend throughout. We suggest reporting statistical information in the following format: ‘x’; (95% UI [‘y’,’ z’] p value) For p values, please report as p<0.001 and where higher as 'p=0.002'. Please add the p value statistical method used to your method section.*

We thank the editor for this request. We have now provided p-values for all estimates of differences between the modeled strategies. The p-value method is included in the Methods on p.19 lines 87-89:

“Point estimates and 95% UIs were also calculated for the differences between leading 6 month BPaLM-based and SOC-based strategies, and p-values were constructed from the empirical cumulative distribution function of those differences.”

Further detail is available in the Supplement p.10 lines 136-143.

*For in-text reference, citation reference numbers are placed within square parentheses and these should precede punctuation. Please amend throughout.*

We have implemented this change.

*STUDY DESIGN:
MODELLING STUDIES
(For the Microsimulation Model)

Of all authors who submit a modelling study we ask for inclusion of specific items, derived from Geoffrey P Garnett, Simon Cousens, Timothy B Hallett, Richard Steketee, Neff Walker. Mathematical models in the evaluation of health programmes. (2011) Lancet DOI:10.1016/S0140-6736(10)61505-X.

Please ensure all the items listed below are included with your manuscript. Please review the list below and confirm/revise as necessary:

(i) Please provide a diagram that shows the model structure, including how the disease natural history is represented, the process and determinants of disease acquisition, and how the putative intervention could affect the system.*

We have updated Figure S4 in response to this request.

*(ii) Please provide a complete list of model parameters, including clear and precise descriptions of each parameter, together with the values or ranges for each, with justification or the primary source cited, and important caveats about the use of these values noted.*

Please see Table S1 and new Table 2.  *(iii) Please provide a clear statement about how the model was fitted to the data.*

We did not calibrate the model per se, but we described how we validated the modeled results in the Methods section.  *(iv) For uncertainty analyses, please state the sources of uncertainties quantified and not quantified.*

Please see the “Distribution” column in Table S1.  *(v) Please provide sensitivity analyses to identify which parameter values are most important in the model. Uncertainty estimates seek to derive a range of credible results on the basis of an exploration of the range of reasonable parameter values. The choice of method should be presented and justified.*

Please see Results section and Figure 5.

*(vi) Please discuss the scientific rationale for this choice of model structure and identify points where this choice could influence conclusions drawn. Please also describe the strength of the scientific basis underlying the key model assumptions.*

We adopted a microsimulation model as we were primarily concerned with the effects of the interventions within the individuals receiving treatment. We did not model the downstream consequences of transmission with a dynamic model, but have noted this as a limitation.

*We thank you for including the CHEERS checklist to cover the cost effectiveness part of the analyses.

DATA AVAILABILITY: For studies in which a model is central to the manuscript's findings, as is the case here, authors are responsible for providing the source code needed to replicate the study's findings in a repository (such as GitHub, SourceForge or Bitbucket) or a cloud computing service (such as Code Ocean). Protection of authors’ intellectual property will not be cause for exception. Please explain in the manuscript’s Data Availability Statement how readers can access the shared code.*

We have updated the Data Availability Statement, and are happy to share the data and source code at <https://github.com/lyndonpjames/BPaLM_Moldova> *ABSTRACT: In Abstract Methods and Findings: As above, please quantify the main results (with 95% UIs and p values).*

Please see the revised Abstract.

*In the last sentence of the Abstract Methods and Findings section, please describe the main limitation(s) of the study's methodology.*

Please see the revised Abstract. *AUTHORS SUMMARY:
At this stage, we ask that you include a short, non-technical Author Summary of your research to make findings accessible to a wide audience that includes both scientists and non-scientists. The Author Summary should immediately follow the Abstract in your revised manuscript. This text is subject to editorial change and should be distinct from the scientific abstract. Please see our author guidelines for more information:* [*https://journals.plos.org/plosmedicine/s/revising-your-manuscript#loc-author-summary*](https://journals.plos.org/plosmedicine/s/revising-your-manuscript#loc-author-summary)

*Ideally each sub-heading should contain 2-3 single sentence, concise bullet points containing the most salient points from your study.

In the final bullet point of ‘What Do These Findings Mean?’ Please include the main limitations of the study in non-technical language.*

Please see Author Summary. We are also very happy to expand on the three bullets per sub-heading if desired. *ACKNOWLEDGMENTS/ DECLARATIONS
Please remove all statements apart from acknowledgements, author contributions and abbreviations from the end of the main manuscript and include these only in the relevant parts of the manuscript submission form. Funding, competing interest, and data availability will be compiled as metadata.*

We have incorporated this change.

*REFERENCES:
Please use the "Vancouver" style for reference formatting, and see our website for other reference guidelines* [*https://journals.plos.org/plosmedicine/s/submission-guidelines#loc-references*](https://journals.plos.org/plosmedicine/s/submission-guidelines#loc-references) *As mentioned above, for in-text reference, citations are placed within square parentheses and should precede punctuation. Please amend throughout.*

We have incorporated this change. *Please also ensure that any references to online-only sources includes a date of accession. Please use [date accessed: ] format instead of ‘cited’. Thank you.* 

We have incorporated this change.

*FIGURES and TABLES;
Please ensure you re-define abbreviations in the legends of figure and tables (e.g Figure 1, 2, 3, etc - Table 2).*

We have done so in all legends.

*Comments from the reviewers:*

*Reviewer #1:*

*Thank you for the opportunity to review the manuscript by James and colleagues, addressing the cost-effectiveness of treatment and drug susceptibility testing strategies for persons with rifampicin-resistant TB in Moldova.  The study findings suggest that adoption of the 6-month BPaLM regimen will be cost-effective compared to the previous standard regimens: in the long term, there are likely to be cost savings, with relatively similar quality-adjusted survival.

Major Comments:
1.      There are several strengths in this analysis, including its use of Moldovan data with respect to evolving drug susceptibilities.  The modeling approach and assumptions are reasonable and generally well described, with suitable sensitivity analyses.  Many of the potential limitations are appropriately acknowledged.*

Many thanks to the reviewer for this feedback.

*2.      That said, there are some areas for clarification and comment.  The first relates to the analysis of quality-adjusted survival.  As this is the major health outcome considered, the assumptions regarding QALY weights, and their justification, need to be included in the main text and tables.  In the current version of the manuscript, this is mentioned in a cursory manner (p. 7, lines 66-68) with an incorrect reference.  There is more information available in the supplement, but key elements of this including suitable explanation and references should be moved to the main text.*

We are grateful to the reviewer for this helpful suggestion. We have amended the text as follows:

p.16 lines 15-26.

“The primary health outcome was measured in quality-adjusted life years (QALYs), a conventional approach in cost-effectiveness analysis [1,2]. QALYs measure the total length of time lived, weighted by time-varying, health-related quality of life, where 1 QALY is valued equivalently to one year in a state of perfect health [42, 43]. This approach integrates the effects of the treatment strategies on both length and quality of life. QALYs rely on several assumptions, including risk-neutrality over length of life, and that maximizing total population QALYs—summed across all individuals—is desirable [3]. For each modeled individual in each month, we assigned health-related quality of life weights on a scale from 0 (dead) to 1 (perfect health), and multiplied the weight by 1/12 to account for the one-month cycle length (i.e., a dead individual accrued 0 QALYs for that month, and an individual in perfect health accrued 1/12 QALYs). These estimates were lowest on average at presentation and increased during the course of treatment; SAEs also conferred a decrement in the utility weight (Table S1). The total QALYs were calculating by summing all the month-specific QALYs accrued over each modeled individual’s lifetime. ”

We also note the citation in question as being incorrect–Weinstein et al., 1977 [43]–was intended to provide a canonical reference on calculating QALYs from their components, but it may not have been appropriate on its own. We welcome any additional feedback on this citation and are happy to remove it altogether if preferred, but hope it is more appropriate alongside the other citations in this paragraph.

*3.       Also related to the QALY outputs, in sensitivity analysis the leading BPaLM strategy was led to an expected decrement of 0.55 QALYs when the hazard rate ratio for cure with that regimen was 1.2 relative to the standard RR-TB regimen.   This is described as "small, non-significant"  [p. 15. Line 33].  I suspect many readers would disagree and I would suggest that this description be dropped.  Perhaps more importantly, the reasons for this non-trivial decrement were not clear—it occurs despite improved cure rates with BPaLM [i.e. with HRR remaining at 1.2].  It did not seem to be due to an increased frequency of severe adverse events.  Even in the base case analysis, BPaLM was associated with a slight decrement in quality-adjusted survival, despite a more marked assumed improvement in cure rates.  Why is this?*

We thank the reviewer for this important observation, which we believe has improved how we communicate important results and features of the model.

First, we removed the descriptor “small, non-significant” as suggested.

Regarding the reasons for the decrement, this comes down to the tradeoff between the enhanced monthly cure rate (which benefits BPaLM, all else equal) and the duration of treatment (benefits SOC, all else equal, as those individuals are exposed to the enhanced monthly cure rate for longer). In order to improve clarity, we have replaced “cure rate” with “monthly cure rate” throughout.

Related to this comment, we also note the editor’s comment about the apparent discrepancy between our model outcomes and the broader findings of TB-PRACTECAL, and think the following will shine some additional light in response to the reviewer’s comment.

The TB-PRACTECAL trial’s main takeaway is derived from its primary outcome, which is a composite measure of death, failed by treatment, LTFU, recurrence, and early discontinuation, measured at 72 weeks (i.e., 17 months). The HRR for culture conversion was measured at 24 weeks (i.e., 6 months).

In the TB-PRACTECAL main results table, it is noteworthy that the largest difference in the modified intention-to-treat analysis came from early discontinuation, especially discontinuation secondary to SAEs and withdrawal of consent. Our model is not capable of capturing all the events in the trial primary outcome (such as withdrawal of consent), and we were primarily interested in estimating long-term outcomes which are not possible in a trial. However, to illustrate the alignment between our results and the trial, we tracked a composite unfavorable outcome similar to that in TB-PRACTECAL at 6 months, 12 months and 72 weeks (i.e., 17 months) (Table S5).

We see that our estimate for the reduction in unfavorable outcome of 6 percentage points (95% UI: [0, 6] p=0.05) aligns quite well with the trial’s per protocol estimate for BPaLM of a reduction of 9 percentage points (95% CI: [reduction 22, gain 4]; see bottom right cell of Table 2 in TB-PRACTECAL).

*4.      Some of the key model input parameters (table S1) should be moved to a table accompanying the main text—the most important natural history, treatment-related, QALY, and cost parameters, for example.   The text in the main methods section outlining cost assumptions should point to the appropriate table(s) [p. 8, lines 85-93].*

Please see page 13 for the requested new table (Table 2).

*5.      The accounting for direct non-medical and indirect costs of RR-TB and its treatment (items 64 and 65) in table S1 is a bit confusing.  The point estimates are listed as $2,044 and $2,301 respectively.  If I have understood correctly, these are based on an 18-month treatment episode, and then converted to monthly estimates.  So for individuals who receive 6 months of treatment, are the point estimates 1/3 of those amounts?  And for 9 months, are they half?*

We thank the reviewer for this observation and accompanying questions.

We have added clarifications to the parameter name column (Table S1) that these are for an 18-month period.

The reviewer’s understanding is correct: The estimates are adopted as the costs for an 18 month treatment episode. As such, an individual who has an uninterrupted treatment course of 6 months would accrue 1/3 of both cost parameters (i.e., they accrue 1/18 of the value per month). An additional clarification is that the indirect cost is accrued not only by all those receiving treatment, but also by those who still have TB but are no longer receiving treatment (e.g., because they have either been LTFU or because they were not truly cured when they were discharged from treatment).

*6.      The identification of dominated vs. non-dominated strategies is based on point estimates for QALYs that fall very close together.  The same holds true for cost estimates for the various antibiotic replacement and susceptibility testing strategies, when the initial treatment regimen is held constant.  There is huge overlap between the various uncertainty ranges.  This at least deserves comment.  It is certainly most efficient, given limited space, to focus on what appear to be the "leading" BPaLM and "conventional treatment" strategies, but differences between these and the "dominated" strategies should not be overstated.*

We thank the reviewer for this observation. We agree that some of the strategies do have considerable overlap. It is for this reason we thought it was very important to include the cost-effectiveness acceptability curve as Figure 1B, which shows the probability of each strategy being the optimal strategy, for different values of willingness-to-pay for health. We note that there is a sizeable chance that any of Strategies 1, 2, and 5 — those where individuals who have to stop moxifloxacin have their regimen “topped back up” to four drugs with clofazimine — is cost-effective.

As noted in the figure legend, strategies were excluded from Figure 1B if they were not cost-effective in any of the 1,000 simulations of the probabilistic sensitivity analysis, and these correspond to strategies 3, 4, and 6 — those where individuals who have to stop moxifloxacin receive BPaL only.

We also hope the inclusion of the following in the text aids in the communication that one of Strategies 1, 2, and 5 is likely to be optimal, rather than Strategy 1 only:

p. 24 Lines 17-18:

“Taken together, the probability that one of these strategies would be most cost-effective was at least 93% across the range of cost-effectiveness thresholds for Moldova.”

Based on the reviewer’s recommendation, we have also changed the text:

p. 24 lines 19-20:

“For simplicity, we henceforth make comparisons between the leading (i.e., most cost-effective based on point estimates) 6-month BPaLM-based and standard of care-based strategies: Strategy (1) and Strategy (7), respectively”

In the Figure legend for 1A, we also provided the following clarification:

p. 23 line 19: “The efficient frontier (black lines) connects the non-dominated strategies based on point estimates.”

In the discussion, we also clarified with the following:

p. 30 lines 6-10: “Though there is considerable overlap between some of the 6-month BPaLM implementation scenarios, there is a clear lead for strategies where clofazimine is used to “top up” the regimen if moxifloxacin must be discontinued because of an SAE or resistant DST result, compared to continuing on the three-drug BPaL regimen alone”

*7.      For this reason, it would be appropriate to show standard cost-effectiveness planes, showing the various point estimates for incremental costs and QALYs from probabilistic analysis, comparing strategies 1 and 7.  Some of this information is of course implicit in figure 5, but it should be shown explicitly in a separate figure.*

We agree with this recommendation. We have produced the incremental cost-effectiveness plane for Strategy 1 as compared to the status quo of Strategy 7 and include this as Figure S7.

*8.      The authors appropriately acknowledge that they have attributed hazard rate ratios for cure for BPalM based on those observed for sputum culture conversion in the TB-PRACTECAL study.  They recognize that sputum culture conversion may be an imperfect surrogate for cure.  With that in mind, it would be useful to extend the sensitivity analysis around  the HRR for cure down to a value of 1, i.e. BPaLM exactly equivalent to standard treatment with respect to cure rate. This would further address uncertainty related to extrapolation of cure rates from sputum conversion rates.*

We thank the reviewer for this suggestion. We extended the lower bound of the sensitivity analysis on the HRR for cure to 1.

This is now visible in Figure 5 and in the Results section, p.28 lines 102-103:

“For low values of the HRR (HRR = 1), Strategy (1) was estimated to lead to a mean 0.90 reduction in QALYs. For high values (HRR = 2), Strategy (1) would lead to a mean 0.35 gain in QALYs.”

*9.      Figure 4 could potentially be moved to the supplement.*

We would be very happy to do so if the reviewer or editor would strongly prefer this. For now, we have kept it as a main Figure because reducing the frequency and severity of severe adverse events is a motivator for improving treatment in RR-TB.

*10.     The modeling approach used here does not account for secondary transmission of RR-TB, and its downstream impacts.  Given the scope and nature of the research question, this is not a major limitation in my view, but it should be acknowledged explicitly in the discussion section.*

We thank the reviewer for this comment. We have edited the discussion as requested:

p. 33 lines 85-91:

“Finally, we did not account for the secondary effects resulting from onward transmission of RR-TB, and our results may therefore not capture the full cost-effectiveness implications of each modelled strategy. To account for this explicitly, it would be necessary to model the transmission dynamics of *M. tuberculosis*. Instead, we estimated the cumulative incidence and duration of resistance as surrogates for the long-term health outcomes they may affect, insofar as lower incidence and fewer months of resistant disease might each result in less transmission of resistant strains.”

*Minor Comments:
1.      Page 11, line 21 and figure 1B:  The probabilities shown are of the strategy in question being the MOST cost-effective, compared to the others—not just the probability of being cost-effective according to the willingness-to-pay threshold shown.   Please change the description in the text, figure legend and labels accordingly.*

We have edited the text to “most cost-effective” throughout in response to this suggestion.

*2.      Table 1:  would be clearer to state "Every 4 months" or "Every 1 month" under "Routine frequency of subsequent DST."*

We have amended Table 1 as suggested.

*Reviewer #2:*

*In addressing the complexities of treating Rifampicin-Resistant Tuberculosis (RR-TB), the WHO's 2022 guidelines introduced a shorter, 6-month BPaLM regimen, contrasting with the earlier 2020 guidelines which recommended longer treatments spanning 9-18 months. This study employed a detailed mathematical model to estimate the long-term health outcomes and costs of these strategies in Moldova, a country with a high TB drug resistance burden. Through the utilization of genomic sequencing and a Markov microsimulation model, the research evaluated regimen effectiveness, potential severe adverse events, and resistance acquisition. The results indicated that the shorter BPaLM regimen could decrease lifetime costs by $3419 per individual while maintaining comparable health outcomes. Moreover, it showcased potential cost-effectiveness, especially in settings where longer treatments are financially and logistically challenging, with minimal implications on drug resistance.

Overall, this is a well-conducted study. However, I noticed some vital aspects of RR-TB that seem overlooked. Below are my specific comments:

1. Line 19-24, Introduction: The introduction mentions concern about the emergence of drug resistance, especially in settings with limited capacity to detect resistance to newer antitubercular agents. However, it might have been insightful to briefly elaborate on why such limitations exist. Is it due to a lack of technology, insufficient training, or some other factors? Offering a glimpse into these challenges could help readers appreciate the complexities involved in implementing new regimens in different global settings.*

We thank the reviewer for this helpful suggestion and have edited the text to the following, incorporating additional citations on the reasons for limited capacity to detect resistance:

p.6 lines 28-32: “Implementation may also be met with concern over the emergence of drug resistance, particularly in settings with limited capacity to detect resistance to newer agents such as bedaquiline, pretomanid, and linezolid [4]; such capacity constraints are multifactorial, from the expense of investing in new technologies and associated laboratory workforce development, to supply chain interruptions and divergent political priorities [5,6].“

*2. Introduction: While the introduction thoroughly covers the clinical and logistical challenges of RR-TB treatment regimens, it lacks a direct reference to the patient's experience. The implications of prolonged treatments, drug toxicity, and the psychological toll of longer therapeutic courses could have been briefly highlighted to provide a more holistic perspective.*

We would like to thank the reviewer for this very helpful insight, and we agree that incorporating the patient’s perspective would enhance the introduction. We have edited the text to reflect this.

p. 5 lines 18-23: “The pursuit of effective shorter treatment regimens is also driven by the desire to alleviate the considerable psychological and emotional toll of prolonged treatment for RR-TB. On top of drug side effects [7], many patients undergoing treatment for RR-TB experience stigma, depression, loss of self-esteem, and economic hardship from an inability to work [8]. Patients may lack access to sufficient psychological and financial supports [9–11], and this may be particularly hard for individuals with housing or employment instability, or substance use disorder [12].”

*3. Line 42-44, Introduction: While the study focuses on Moldova, a country with a high burden of TB drug resistance, the introduction does not sufficiently contextualize Moldova's unique challenges, healthcare infrastructure, or previous treatment efforts. Such contextual information could offer insights into why Moldova is particularly relevant for this research, ensuring readers understand the significance of the chosen setting.*

We thank the reviewer for this comment, and have expanded the introduction to reflect this.

Page 7, lines 53-63:

“We conducted the analysis for the setting of Moldova, an upper-middle income post-Soviet country where the incidence rate of RR-TB is among the highest in the world, and where an estimated 33% of individuals newly diagnosed with TB have RR-TB, ten times higher than the same proportion globally [13,14]. The reasons for this picture are not fully understood, but it is thought that economic shocks following the breakup of the Soviet Union contributed to this picture in the region, along with early treatment discontinuation [15] and mass incarceration [16]. In recent years in Moldova, a multidisciplinary committee reviews the treatment course of every patient receiving treatment for RR-TB, and WHO treatment guidelines are closely adhered to [VC, DC]. Moldova also has developed a robust TB laboratory infrastructure, which provided a platform for recent genomic sequencing of culture-positive isolates [17]. By harnessing this genomic resistance data, we hope to improve the cost-effectiveness of treatment in a country with a very high burden of RR-TB, as well as other countries in the region.”

*4. Line 25-28, Method: The two assumptions mentioned here are pivotal to the simulation results. It's essential to validate these assumptions through consultation with field experts to ensure accuracy and relevance.*

Thanks to the reviewer for this helpful comment, which referred to the following statement regarding assumptions in the original submission: “We assumed that there was only one strain of M. tuberculosis per individual (i.e., that there were no mixed infections) and that a mutation associated with resistance conferred full resistant to that drug. M. tuberculosis strains lacking relevant resistance mutations were assumed to be fully susceptible to the respective drugs”

First, on the assumption of there being no mixed infections, below we detail the important ways this assumption plays out, and how we have addressed each:

- The genomic sequencing data from Moldova specifically excluded mixed infections: Of 2,220 samples, 386 mixed samples were excluded (17.4%) [17]. Author and field expert TC was the senior author of that analysis. Because we simulated our modeled cohort from that dataset, a limitation is that our conclusions may not address the subset of patients in Moldova with mixed infections. We have added this to the Discussion section, see p.32 lines 56-60:
  “Because the publicly-available WGS dataset excluded samples with mixed strains of *M. tuberculosis* (17.4%), it is possible that our findings do not adequately address this subpopulation with mixed infections, although we note that all the remaining model parameters reflect the real-world health outcomes and costs of a mix of mono- and mixed-strain infections. “
- In clinical care where there is no access to genomic sequencing, it is in almost all situations unknown whether an individual has a mixed infection or not. For example, if a true resistance is detected by phenotypic or molecular testing, this could be a feature of the single strain in a case of mono-infection, or a feature of any or all strains in the case of a mixed infection. Apart from the genomic sequencing dataset, all other model parameters—including those most pertinent to the comparative effectiveness and costs of each regimen—are thus an average between those with and without mixed infections. Prompted by the reviewer’s helpful comment to reflect on our wording, we thus no longer think it is appropriate to say that we *assume* there are no mixed infections. Instead, the *limitation* is an imperfect match between the data used to simulate the population (excludes mixed infections) and the model’s structure and all other parameters (consistent with real world clinical events and outcomes, which include mixed infections). We thus removed this as an assumption from the Methods section. The limitation sentence is provided in the previous bullet
- We edited the figure legend for the exclusion criteria flow diagram (Figure S2) to make it more clear that mixed infections were excluded from the dataset upstream of our analysis: “*The full dataset did not contain any samples with mixed strains of M. tb.”
- We also note that the deconvolution of drug-resistant strains within mixed strain infection samples is an area of current research [18], but is complex and currently not ready to implement in clinical decision making

Second, we respond to the comment on the assumption that a mutation associated with resistance confers full resistance to that drug. This is an area of current research, such as in this recent analysis from the CRyPTIC Consortium [19]. We point in particular to their Table 2, which shows that compared to the gold standard of phenotypic DST, genomic sequencing has very good test characteristics for isoniazid, rifampicin, and ethambutol, but sensitivity is lower for moxifloxacin, amikacin, and ethambutol. As such, we have added this as a limitation, see p.32 lines 60-64:
“we assumed that that the true resistance profile was perfectly predicted by the presence or absence of mutations conferring resistance in this data: While the sensitivity and specificity of genomic sequencing is very high for detecting resistance in rifampicin, isoniazid, and ethambutol, the performance is less favorable for moxifloxacin, amikacin, and ethionamide [19].”

*5. Line 35-36, Method: Is the simulation of individuals based on the 674 distinct samples referenced in line 29? The authors might consider clarifying this point for better understanding.*

Thanks to the reviewer for this clarifying observation. We had amended the text.

p.11 lines 8-9:

“We excluded data for rifampicin susceptible strains (Fig S2) leaving 674 distinct samples from which we simulated the modeled population”

*6. Line 37-38, Method: Is spontaneous recovery from TB a possible state to consider?*

We do model the event of spontaneous recovery (i.e., self-cure) from TB, although we do not model this as a separate health state. However, individuals who have spontaneously recovered are tracked in the model as such at the individual level, and so the subsequent event probabilities, health rewards and costs are accumulated in the same way as if we had modeled with a separate health state.

To clarify, we also amended the text on p.11 lines 18-19:

“Within each Markov state, individual events were tracked including true cure as a result of treatment or self-cure…”

*7. Line 45, Method: It would be beneficial to detail grade 4-5 SAE in the main text, enhancing clarity for readers. Moreover, if a categorized list of SAE by grade is available, assigning these grades to individuals based on the likelihood of each grade's occurrence could refine the results for greater accuracy.*

Thank you, and we have clarified by editing “SAE” to “grade 4-5 SAE” in the main text from Table 1 onwards.

Though we agree with the reviewer that the results would be refined by modeling the risks, costs and consequences by grade of SAE, unfortunately the published estimates did not permit the categorization of SAE by grade 4 vs. grade 5.

*8. Method: While the authors' emphasis on the RR-TB infected population is clear, the effects of mistreated/untreated RR-TB cases on the general susceptible population cannot be ignored (e.g., TB transmission) when considering which strategies are more effective than others. So, the study's results may present only a subset of the full implications regarding the cost-effectiveness of the assessed strategies.*

We thank the reviewer for this observation. As for our response to Reviewer 1’s Major comment #10 (above), we have edited the discussion to state this more clearly:

p. 33 lines 85-91:

“Finally, we did not account for the secondary effects resulting from onward transmission of RR-TB, and our results may therefore not capture the full cost-effectiveness implications of each modelled strategy. To account for this explicitly, it would be necessary to model the transmission dynamics of *M. tuberculosis*. Instead, we estimated the cumulative incidence and duration of resistance as surrogates for the long-term health outcomes they may affect, insofar as lower incidence and fewer months of resistant disease might each result in less transmission of resistant strains.”

*9. Method: Additionally, the omission of HIV infection, a significant risk factor for RR-TB, from the study's considerations is a substantial limitation to me.*

We thank the reviewer for this remark and we have added this limitation to the discussion while acknowledging that this is an area of uncertainty and complexity:

p. 33 lines 81-84:

“While the relationship between HIV and RR-TB treatment outcomes is neither straight-forward nor consistent [20,21], we did not model HIV status at the individual level and as such we were unable to comment specifically on health outcomes for those with TB-HIV coinfection.”

*Reviewer #3:*

*With interest I have read "Impact and cost-effectiveness of the 6-month BPaLM regimen for rifampicin-resistant tuberculosis: a mathematical modeling analysis"

*       Abstract, line 35-37, The authors should clarify the objective of the study. Include which study outcomes were compared.*

Thank you for this helpful comment. We have edited the Abstract to reflect this.

p. 1 lines 39-42:

“The primary objective was to determine whether 6 months of BPaLM is a cost-effective treatment strategy for RR-TB. We used genomic and demographic data to parameterize a mathematical model estimating long-term health outcomes measured in quality-adjusted life years (QALYs) and lifetime costs in 2022 USD ($)”

**       Methods line 91, how were direct non-healthcare costs and indirect costs collected?*

We thank the reviewer for this question. These estimates were adopted from published estimates by Portnoy et al. [22], in which the authors used a meta-regression approach to produce cost estimates for these categories for each of 135 low- and middle-income countries, including the Republic of Moldova (Portnoy et al. Tables S9 and S10). We cite this in Table S1 (parameters 65 and 66) and we have also now clarified this in the text, p.18 lines 53-54:

“Direct non-medical and indirect costs were informed by published estimates for Moldova [22].”

**       Discussion: The authors should explain why their projections show better health outcomes, although small, for the SoC vs the BPaLM strategies, given that the reverse was found in the TB-PRACTECAL trial in which BPaLM showed favourable efficacy when compared with the regimens given in the control arm.*

We thank the reviewer for this very helpful observation.

The TB-PRACTECAL trial’s main takeaway was derived from its primary outcome, which is a composite measure of death, failed by treatment, LTFU, recurrence, and early discontinuation, measured at 72 weeks (i.e., 17 months).

In the TB-PRACTECAL main results table, it is noteworthy that the largest difference in the modified intention-to-treat analysis came from early discontinuation, especially discontinuation secondary to SAEs and withdrawal of consent. Our model is not capable of capturing all the events in the trial primary outcome (such as withdrawal of consent), and we were primarily interested in estimating long-term outcomes which are not possible in a trial. However, to illustrate the alignment between our results and the trial, we tracked a composite unfavorable outcome similar to that in TB-PRACTECAL at 6 months, 12 months and 72 weeks (i.e., 17 months) and are happy to include this in the revised manuscript as Table S5.

We see that our estimate for the reduction in unfavorable outcome of 6 percentage points (95% UI: [0, 6] p=0.05) aligns quite well with the trial’s per protocol estimate for BPaLM of a reduction of 9 percentage points (95% CI: [reduction 22, gain 4]; see bottom right cell of Table 2 in TB-PRACTECAL). As we modeled strategies per-protocol, we think this is likely a better comparison than to the trial’s modified intention-to-treat estimate.

**       Study objective: Considering that WHO is already recommending the BPaLM regimen since 2022 the question is whether addressing it's cost-effectiveness compared to SoC as described in this manuscript is the most relevant outcome measure. For NTPs planning to introducing or scaling-up this regimen it may be more informative to understand the budget impact vs SoC, and whether it would likely result in cost-savings or would require additional budget. The authors could show the budget impact for Moldova.*

We are happy to incorporate a budget impact analysis with the revised manuscript. Please see Methods p.18, lines 74-81, and Table S6.

**       Avoid using stigmatizing language such as 'TB case', instead use 'person with TB'*

We thank the reviewer for this important remark. We intended not to use such stigmatizing language. We edited the text throughout.

References

1.	Hunink MGM. Decision making in health and medicine:integrating evidence and values. Second edition. Cambridge: University Press; 2014.

2.	Weinstein MC, Stason WB. Foundations of Cost-Effectiveness Analysis for Health and Medical Practices. N Engl J Med. 1977;296: 716–721. doi:10.1056/NEJM197703312961304

3.	Weinstein MC, Torrance G, McGuire A. QALYs: The Basics. Value Health. 2009;12: S5–S9. doi:10.1111/j.1524-4733.2009.00515.x

4.	Vanino E, Granozzi B, Akkerman OW, Munoz-Torrico M, Palmieri F, Seaworth B, et al. Update of drug-resistant tuberculosis treatment guidelines: A turning point. Int J Infect Dis IJID Off Publ Int Soc Infect Dis. 2023; S1201-9712(23)00089–9. doi:10.1016/j.ijid.2023.03.013

5.	Practical manual on tuberculosis laboratory strengthening, 2022 update. [cited 21 Nov 2023]. Available: https://www.who.int/publications-detail-redirect/9789240061507

6.	Pai M, Palamountain KM. New tuberculosis technologies: challenges for retooling and scale-up [State of the art series. New tools. Number 4 in the series]. Int J Tuberc Lung Dis. 2012;16: 1281–1290. doi:10.5588/ijtld.12.0391

7.	Lan Z, Ahmad N, Baghaei P, Barkane L, Benedetti A, Brode SK, et al. Drug-associated adverse events in the treatment of multidrug-resistant tuberculosis: an individual patient data meta-analysis. Lancet Respir Med. 2020;8: 383–394. doi:10.1016/S2213-2600(20)30047-3

8.	Thomas BE, Shanmugam P, Malaisamy M, Ovung S, Suresh C, Subbaraman R, et al. Psycho-Socio-Economic Issues Challenging Multidrug Resistant Tuberculosis Patients: A Systematic Review. PLOS ONE. 2016;11: e0147397. doi:10.1371/journal.pone.0147397

9.	Isaakidis P, Rangan S, Pradhan A, Ladomirska J, Reid T, Kielmann K. “I cry every day”: experiences of patients co-infected with HIV and multidrug-resistant tuberculosis. Trop Med Int Health TM IH. 2013;18: 1128–1133. doi:10.1111/tmi.12146

10.	Morris MD, Quezada L, Bhat P, Moser K, Smith J, Perez H, et al. Social, economic, and psychological impacts of MDR-TB treatment in Tijuana, Mexico: a patient’s perspective. Int J Tuberc Lung Dis Off J Int Union Tuberc Lung Dis. 2013;17: 954–960. doi:10.5588/ijtld.12.0480

11.	Mauch V, Melgen R, Marcelino B, Acosta I, Klinkenberg E, Suarez P. Tuberculosis patients in the Dominican Republic face severe direct and indirect costs and need social protection. Rev Panam Salud Publica Pan Am J Public Health. 2013;33: 332–339. doi:10.1590/s1020-49892013000500004

12.	Kendall EA, Theron D, Franke MF, van Helden P, Victor TC, Murray MB, et al. Alcohol, hospital discharge, and socioeconomic risk factors for default from multidrug resistant tuberculosis treatment in rural South Africa: a retrospective cohort study. PloS One. 2013;8: e83480. doi:10.1371/journal.pone.0083480

13.	Global tuberculosis report 2020. [date accessed: 4 Apr 2023]. Available: https://www.who.int/publications/i/item/9789240013131

14.	Yang C, Sobkowiak B, Naidu V, Codreanu A, Ciobanu N, Gunasekera KS, et al. Phylogeography and transmission of M. tuberculosis in Moldova. 2021 Jul p. 2021.06.30.21259748. doi:10.1101/2021.06.30.21259748

15.	Download: The New Profile of Drug-Resistant Tuberculosis in Russia: A Global and Local Perspective: Summary of a Joint Workshop by the Institute of Medicine and the Russian Academy of Medical Science | The National Academies Press. [cited 21 Nov 2023]. Available: https://nap.nationalacademies.org/download/13033

16.	Stuckler D, Basu S, McKee M, King L. Mass incarceration can explain population increases in TB and multidrug-resistant TB in European and central Asian countries. Proc Natl Acad Sci. 2008;105: 13280–13285. doi:10.1073/pnas.0801200105

17.	Yang C, Sobkowiak B, Naidu V, Codreanu A, Ciobanu N, Gunasekera KS, et al. Phylogeography and transmission of M. tuberculosis in Moldova: A prospective genomic analysis. PLOS Med. 2022;19: e1003933. doi:10.1371/journal.pmed.1003933

18.	Wang L, Campino S, Phelan J, Clark TG. Mixed infections in genotypic drug-resistant Mycobacterium tuberculosis. Sci Rep. 2023;13: 17100. doi:10.1038/s41598-023-44341-x

19.	The CRyPTIC Consortium. A data compendium associating the genomes of 12,289 Mycobacterium tuberculosis isolates with quantitative resistance phenotypes to 13 antibiotics. Ladner J, editor. PLOS Biol. 2022;20: e3001721. doi:10.1371/journal.pbio.3001721

20.	Sergeev R, Colijn C, Murray M, Cohen T. Modeling the dynamic relationship between HIV and the risk of drug-resistant tuberculosis. Sci Transl Med. 2012;4: 135ra67. doi:10.1126/scitranslmed.3003815

21.	Bastos ML, Lan Z, Menzies D. An updated systematic review and meta-analysis for treatment of multidrug-resistant tuberculosis. Eur Respir J. 2017;49. doi:10.1183/13993003.00803-2016

22.	Portnoy A, Yamanaka T, Nguhiu P, Nishikiori N, Garcia Baena I, Floyd K, et al. Costs incurred by people receiving tuberculosis treatment in low-income and middle-income countries: a meta-regression analysis. Lancet Glob Health. 2023;11: e1640–e1647. doi:10.1016/S2214-109X(23)00369-8
