# Supplementary material for: Impact and cost-effectiveness of the 6-month BPaLM regimen for rifampicin-resistant tuberculosis in Moldova: A mathematical modeling analysis
Source: PLoS Med. 2024 May 3;21(5):e1004401. doi: 10.1371/journal.pmed.1004401 (PMC11101189; doi:10.1371/journal.pmed.1004401)
Supplement: Attachment — Submitted filename: PLOSMed_AcceptedComments_20240313.docx [file pmed.1004401.s020.docx]

*Comments from the academic editor

To ensure the readers are aware that these data should only be generalised to other settings with high RR background rates and similar income level and that further impact and CE research is needed. This can be done by adding more detail to the study setting in:

(1) the title "Impact and cost-effectiveness of the 6-month BpALM regimen for rifampicin-resistant tuberculosis IN MOLDOVA: a..."*

We thank the editor for this comment and have implemented this change.

*(2) the abstract conclusion: currently says "particularly in settings where current long-course regimens are challenging to implement and afford. Further research may be warranted to explore the suitability of shorter RR-TB regimens in specific national settings"

but not sure whether long course regimen implementation and affordability challenges are the generalisable factors. Would rephrase to

"particularly in settings with a high burden of drug-resistant TB. Further research may be warranted to explore the impact and CE of shorter RR-TB regimens across settings with varied drug resistant TB burden and national income levels."*

We thank the editor for this comment and have implemented this change.

------------------------------------------------------------

*Comments from Reviewers:

Reviewer #1: Thank you for the opportunity to re-review this very well-written manuscript.  And thank you to the authors for their extensive and careful revisions.  I have identified three areas where I believe further revision/clarification would be helpful.

1.      I think the main manuscript would be further strengthened by the addition of a few key QALY and cost parameters to table 1.  I realize that there are many parameters and assumptions within each category, but a few key or high-level examples would be helpful to readers.  For example, the QALY decrement associated with an episode of RR-TB disease that is successfully treated with one of the six-month regimens [I recognize that this integrates multiple monthly QALY weights, which are detailed in the supplement].  On the cost side, an example could be the "typical" combined drug costs for each of the main regimens modeled.  Of course, readers who are particularly interested in detailed aspects of the modeling and the parameters used will refer to the supplementary materials, but placing such items in the main manuscript allows all readers to get some of the context and framing around these parameters.*

We thank the reviewer for this comment and agree that the addition of key utility weight and cost parameters would be helpful in the main manuscript.

As such, we have added two additional rows to Table 2* for utility weights, one for the range of utility weights depending on the month of treatment, and another for the decrease in utility weight conveyed by each grade 4-5 SAE. We hope this is a suitable set of utility weight parameters for inclusion; unfortunately we do not think it would be possible to include as an input parameter the requested QALY decrement associated with an episode of RR-TB disease that is successfully treated with one of the six-month regimens, as this is a model output/result. However, we are very happy to continue the discussion on this if the reviewer would prefer.

We also added two rows for cost inputs, one for the range of typical combined drug costs for the regimens used in BPaLM-based strategies, and another for standard of care-based strategies.

We also noticed that we had used both “health-related quality of life weight” and “utility weight” interchangeably throughout the paper, and so for consistency and clarity

we have now adopted “health-related quality of life weight” throughout.

*Here we assume the reviewer was referring to the main manuscript parameter table (Table 2 rather than Table 1) but if we were incorrect on this, we do apologize.

*2.      Along similar lines, the authors' description of QALY weights in the main text (p. 16) was not exactly what I meant in my earlier comments—I apologize for any lack of clarity on my part.  I was hoping that the authors could provide a clearer picture (and perhaps justification) of the QALY weights they attributed to people undergoing treatment for RR-TB.  The added theoretical definition and the discussion of key assumptions underlying the general use of QALYs are interesting for readers, but not essential to the main manuscript, in my view.*

Many thanks to the reviewer for following up with this helpful clarification.

We have extended the description of how we accounted for health-related quality of life weights in the methods section to provide a clearer picture, under the “Model” subheading. Please see page 12, lines 24-30.

We have also moved the theoretical definition and key assumptions underlying the QALY from the “Outcomes” subheading on page 17 to the S1 appendix. The remaining section on page 17, lines 15-20 is a simpler description of how QALYs are calculated as a function of both length and quality of life.

*3.      Perhaps most importantly, I am still struggling to understand the discordance between expected QALYs and favorable outcomes in the primary comparison between the BPaLM [C] and the "standard of care" regimens.  I appreciate the authors' clarification that the hazard ratios refer to monthly cure rates, so one must consider that the standard regimens last substantially longer--even if the monthly cure rate is less.  But the model outputs also include an overall reduction in unfavorable outcomes with the shorter regimens—as found in the TB-PRACTECAL trial—so why are QALYs lower with the shorter regimens?  Again, this does not appear to reflect adverse events.  I believe this discordance requires clarification and specific explanation in the text.  If there is an explanation which I have missed, I apologize.*

We agree with the reviewer that this may appear to be contradictory and have sought to expand considerably on our previous explanation. In brief, the key contributor is that the trial’s unfavorable outcome measure, and the two unfavorable outcome measures we modeled, are composite outcomes. They include events with potentially very different impacts on long term health as measured in QALYs. For example, let us contrast a) death – carrying a very large QALY impact – with b) an adverse event, having a meaningful but typically much smaller impact on QALYs, and with c) having been failed by treatment, which is deemed to have occurred if at least two drugs had to be changed; the QALY impact could even be beneficial here if the change was in response to the detection of drug resistance and thus a switch to drugs more effective for that individual’s strain of *M. tb*. Under the composite, all three would be counted in the same way as an unfavorable outcome.

Please see page 31, lines 24-41 for our explanation: “When modeling the comparative effectiveness of 6 months BPaLM … while they were captured in QALYs which had a lifetime horizon.” We are happy to expand on this further if desired by the reviewer or editor.

*A minor point:  The Y-axis label in figure 1B should be corrected to read, "Probability of being most-cost-effective."*

We have implemented this change.

*Reviewer #2: In my opinion, the authors have sufficiently addressed my and other reviewers' questions and concerns and thus the paper is acceptable in its current form. Congratulations to the authors!*

We would like to thank the reviewer for these remarks!

------------------------------------------------------------

*Comment from the editorial team*

*1.      Please address the outstanding comments from the academic editor and the reviewer.*

We have addressed the comments above, but we welcome the editor’s feedback as to whether we should make any further improvements.

*2.      Given the study design, please do not use causal language. "Effect" should be used only if causality can be inferred, i.e., for an RCT. Please check and amend through (including the authors’ summary).*

We were happy to amend throughout.

*3.      Data Availability statement. Thank you for agreeing to make your data available. At this time, please provide the direct link to the data file within the repository. Please also add this link to the description of Figure S2, the figure where you describe how you obtained your data set (and the exclusion criteria you applied)*

We were happy to add the relevant links to Figure S2. The data with exclusion criteria applied are present within the .trex model file as “tbl_WGSempiricaldata_RR”.

*4.      Please rephrase sentences that start with a number. (e.g. see line 49 and 51). Please check and amend throughout.*

We were happy to amend throughout.

*5.      Please refer to people/patients as … people/patients with TB or RR-TB instead of (RR-)TB patients.*

We thank the editor for spotting this, and we were happy to amend throughout.

*6.      General comment - supplementary materials: Please note that supplementary materials are not checked and will be posted as supplied by the authors. Therefore, please double check. Please cite your Supporting Information as outlined here:* [*https://journals.plos.org/plosmedicine/s/supporting-information*](https://journals.plos.org/plosmedicine/s/supporting-information) *- Please note you may use almost any description as the item name of your supporting information as long as it contains an "S" and number. For example, “S1 Appendix” and “S2 Appendix,” “S1 Table” and “S2 Table.*

*7.      Can you check that S5 Figure (S5 Fig) has a main body text call-out? It seems to have been omitted, please double check. I have the same comment for S6 Figure (S6 Fig) and (S8 Fig)*

We thank the editor for catching these omissions, and have added call-outs to these three supplementary figures.

*Please do not hesitate to contact me directly at* [*kjanin@plos.org*](mailto:kjanin@plos.org) *if you have any questions about the above

Best wishes,
Katrien*
